# Pre-Bilaterian Origins of the Hox Cluster and the Hox Code: Evidence from the Sea Anemone, *Nematostella vectensis*

**DOI:** 10.1371/journal.pone.0000153

**Published:** 2007-01-24

**Authors:** Joseph F. Ryan, Maureen E. Mazza, Kevin Pang, David Q. Matus, Andreas D. Baxevanis, Mark Q. Martindale, John R. Finnerty

**Affiliations:** 1 Bioinformatics Program, Boston University, Boston, Massachusetts, United States of America; 2 Department of Biology, Boston University, Boston, Massachusetts, United States of America; 3 Kewalo Marine Laboratory, Pacific Bioscience Research Center, University of Hawaii, Honolulu, Hawaii, United States of America; 4 Genome Technology Branch, National Human Genome Research Institute, National Institutes of Health, Bethesda, Maryland, United States of America; Washington University in St. Louis School of Medicine, United States of America

## Abstract

**Background:**

Hox genes were critical to many morphological innovations of bilaterian animals. However, early Hox evolution remains obscure. Phylogenetic, developmental, and genomic analyses on the cnidarian sea anemone *Nematostella vectensis* challenge recent claims that the Hox code is a bilaterian invention and that no “true” Hox genes exist in the phylum Cnidaria.

**Methodology/Principal Findings:**

Phylogenetic analyses of 18 Hox-related genes from *Nematostella* identify putative Hox1, Hox2, and Hox9+ genes. Statistical comparisons among competing hypotheses bolster these findings, including an explicit consideration of the gene losses implied by alternate topologies. *In situ* hybridization studies of 20 Hox-related genes reveal that multiple Hox genes are expressed in distinct regions along the primary body axis, supporting the existence of a pre-bilaterian Hox code. Additionally, several Hox genes are expressed in nested domains along the secondary body axis, suggesting a role in “dorsoventral” patterning.

**Conclusions/Significance:**

A cluster of anterior and posterior Hox genes, as well as ParaHox cluster of genes evolved prior to the cnidarian-bilaterian split. There is evidence to suggest that these clusters were formed from a series of tandem gene duplication events and played a role in patterning both the primary and secondary body axes in a bilaterally symmetrical common ancestor. Cnidarians and bilaterians shared a common ancestor some 570 to 700 million years ago, and as such, are derived from a common body plan. Our work reveals several conserved genetic components that are found in both of these diverse lineages. This finding is consistent with the hypothesis that a set of developmental rules established in the common ancestor of cnidarians and bilaterians is still at work today.

## Introduction

Thousands of papers have been written about Hox genes over the last twenty years, and cross-species comparisons using this particular family of homeodomain transcription factors have provided the critical momentum sparking the recent resurgence of the field of evolutionary developmental biology. Three remarkable phenomena that have fueled enormous interest in Hox genes are the Hox code, Hox clusters, and Hox colinearity.

In a phylogenetically diverse range of animals, a conserved “Hox code” is partially responsible for patterning the primary body axis. The term Hox code was first applied to the segmentally-restricted expression of Hox genes in the branchial system of the developing mouse [1]. However, extensive similarity among Hox expression patterns in a wide range of taxa soon led to the recognition that a Hox code might be a fundamental developmental mechanism of animals [2]. In all bilaterian animals that have been studied, multiple Hox genes are found in their genomes and, over the course of development, different regions along the primary body axis come to express different Hox genes or different combinations of Hox genes. Appropriate Hox expression is required to confer the appropriate regional identity upon these Hox-expressing body regions — *ergo*, a Hox code. Furthermore, comparable body regions are patterned by orthologous Hox genes in distantly related taxa, so a similar Hox code appears to be widely conserved. However, this does not mean that the “Hox code” is static over evolutionary time. Hox expression patterns can vary substantially with respect to how much overlap exists between expression domains, what fraction of the primary body axis is accounted for by Hox expression, the precise axial order of different Hox orthologs, the degree of dorsal-ventral asymmetry in Hox expression, and the germ layer in which Hox genes are expressed. With regards to this last point, while Hox genes are generally regarded as exhibiting ectodermal and mesodermal expression, they are also expressed in endoderm [3]–[10].

In addition, across a range of bilaterian animals, Hox genes are located in conserved genomic clusters. The relative genomic organization of orthologous Hox genes is well conserved among select Ecdysozoa such as *Anopheles*, *Schistocerca*, and *Tribolium* [11]–[14]; Lophotrochozoa, such as *Lineus* [15]; and Deuterstomia, such as vertebrates and *Branchiostoma* [16], [17]. The origin of Hox clusters is not especially remarkable, since the Hox clusters would have arisen as a natural outgrowth of the process of tandem gene duplication. However, the persistence of Hox clusters over hundreds of millions of years in diverse metazoan lineages suggests that strong stabilizing selection must be operating.

One explanation for the conservation of genomic organization is that the proper regulation of these genes may depend upon their close physical linkage (reviewed in [18]. However, in diverse bilaterian taxa (for example, *Ciona intestinalis*, *Caenorhabditis elegans*, *Drosophila melanogaster*, *D. pseudoobscura*, *D. repleta*, *D. virilis*, *Oikopleura dioica*, *Schistosoma mansoni*, and *Strongylocentrotus purpuratus*), the Hox cluster has experienced breaks, undergone extensive rearrangements, or even degenerated to the point where a cluster cannot be recognized or identified [19]–[27]. The degeneration of the Hox cluster does not necessarily imply that the Hox code has been abandoned, as Hox genes may continue to specify the same axial territories even after a Hox cluster has undergone extensive rearrangements. For example, it appears that all insects employ the same Hox code, but some insects have intact Hox clusters (for example, grasshopper), while others have partially degraded Hox clusters (for example, fruit flies).

It has also been observed in a phylogenetically widespread range of taxa that the relative spatial and/or temporal expression of Hox genes is correlated with their relative position within Hox clusters. This correspondence between gene expression and cluster organization has been termed *colinearity* [28]. The existence of colinearity implies that linkage impacts gene regulation [18]. However, Hox colinearity is not universal [29], and no single mechanism has been identified that can explain Hox colinearity or the persistence of Hox clusters in diverse metazoan lineages [30]. Rather, it seems that several different regulatory mechanisms may contribute to the stability of Hox clusters. For example, both higher-order chromatin structure and local *cis*-regulatory elements may result in coordinated regulation of neighboring Hox loci [31], [32]. Furthermore, in some taxa, most notably *Drosophila*, Hox linkage does not appear to be required for appropriate Hox expression [33]. The general correspondence between the genomic organization and spatial expression of Hox genes in *Drosophila* may be attributable to phylogenetic inertia.

Over evolutionary time, the functional diversification of Hox genes has clearly contributed to the diversification of animal body plans [5], [34]–[40]. For this reason, understanding the origin and early evolution of Hox genes could prove critical to understanding the metazoan radiation. A Hox cluster consisting of seven genes evolved prior to the divergence of protostomes and deuterostomes [41] and, as both insects and vertebrates utilize Hox genes to pattern a portion of their primary body axes, the Hox code can be said to predate the diversification of crown bilaterians. The phylum Cnidaria can provide unique insights into early Hox evolution since cnidarians constitute an outgroup to the Bilateria [42], [43].

It is currently a matter of debate whether cnidarians possess *bona fide* Hox genes, and if so, whether the Hox code originated prior to the cnidarian-bilaterian divergence. In the last few years, several studies have suggested that cnidarians possess both anterior and posterior Hox genes, but they lack group 3 and central Hox genes [36], [44]–[48] (see Table 1 for varying gene nomenclature). More recently, Kamm and co-workers have advocated two seemingly contradictory hypotheses: (1) that cnidarians possess anterior Hox genes, but instead of *bona fide* posterior Hox genes, they possess a posterior Hox/Cdx like gene [49], and (2) that cnidarian genes related to bilaterian Hox genes “should be regarded as Hox-like but not as true Hox genes” [50]. A more recent study by Chourrout and co-workers suggests that the cnidarian-bilaterian ancestor possessed two to three ParaHox genes as well as an Anterior and group 3-like Hox gene, each of which subsequently underwent independent radiations within the bilaterian and cnidarian lineages [51].

**Table 1:**
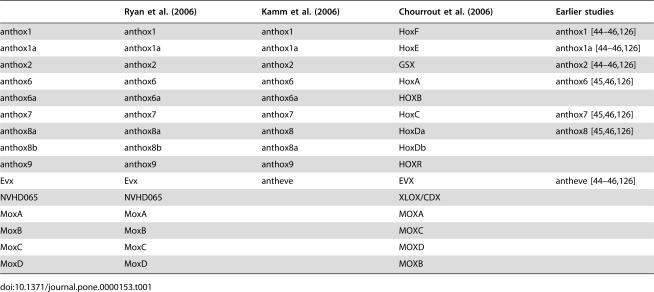
Nomenclature of Nematostella Hox and Hox-related genes.

	Ryan et al. (2006)	Kamm et al. (2006)	Chourrout et al. (2006)	Earlier studies
anthox1	anthox1	anthox1	HoxF	anthox1 [44]–[46], [126]
anthox1a	anthox1a	anthox1a	HoxE	anthox1a [44]–[46], [126]
anthox2	anthox2	anthox2	GSX	anthox2 [44]–[46], [126]
anthox6	anthox6	anthox6	HoxA	anthox6 [45], [46], [126]
anthox6a	anthox6a	anthox6a	HOXB	
anthox7	anthox7	anthox7	HoxC	anthox7 [45], [46], [126]
anthox8a	anthox8a	anthox8	HoxDa	anthox8 [45], [46], [126]
anthox8b	anthox8b	anthox8a	HoxDb	
anthox9	anthox9	anthox9	HOXR	
Evx	Evx	antheve	EVX	antheve [44]–[46], [126]
NVHD065	NVHD065		XLOX/CDX	
MoxA	MoxA		MOXA	
MoxB	MoxB		MOXC	
MoxC	MoxC		MOXD	
MoxD	MoxD		MOXB	

With respect to the Hox code, a 2004 study on the expression of five candidate Hox genes in the anthozoan sea anemone *Nematostella vectensis* found support for the existence of a Hox code in the cnidarian-bilaterian ancestor; multiple Hox genes appeared to be present, and they were found to be expressed in distinct territories along the primary body axis [36]. In contrast, the aforementioned 2006 study by Kamm et al. [49] concluded that the Hox code was a bilaterian invention based on what they regarded as the absence of central, group 3, and posterior Hox genes in Cnidaria, and on differences between the Hox expression patterns between the anthozoan *Nematostella* and the colonial hydrozoan *Eleutheria*.

In the current study, we employ novel analytical methods and present an extensive battery of new evidence from the sea anemone *Nematostella vectensis* to address the origin and early evolution of Hox genes and the Hox code. This evidence includes phylogenetic analysis of eighteen distinct Hox-related loci from *Nematostella* [52], linkage analysis of these eighteen loci based on an assembly of the *Nematostella* genome [53] and extensive corroborating gene mapping studies; and developmental gene expression assays for 20 *Nematostella* Hox-related genes, 12 of which have never been described before. For those genes whose expression has been previously described, we reveal previously unknown aspects of the spatiotemporal expression that are critical to interpreting Hox evolution.

Contrary to some recent reports [49], [50], multiple lines of phylogenetic evidence support the hypothesis that both anterior and posterior Hox genes and two ParaHox genes were present in the cnidarian-bilaterian ancestor. Seven *Nematostella* genes appear to be descended from the founding members of the Hox1, Hox2, and Hox9+ families. We provide more detailed transcriptional annotation of a genomic cluster comprising two ParaHox genes as well as one Hox1 family member, three Hox2 family members, an even-skipped ortholog, an HlxB9 ortholog, and a Rough ortholog [51].

During larval development, the putative Hox1, Hox2, and Hox9+ homologs are expressed in a number of distinct spatial domains that collectively account for practically the entire primary body axis, from the aboral to the oral extremity. Five of these candidate Hox genes (anthox7, anthox8, anthox8a, anthox6a, anthox1a) and one candidate ParaHox gene (NVHD065) are also differentially expressed along the secondary body axis, known as the directive axis. These genes are expressed in nested subsets along the directive axis, suggesting that *Nematostella* may be employing Hox genes to pattern both its primary and secondary axes. Phylogenetic mapping of gene expression patterns on a molecular phylogeny suggests that differential expression along the primary body axis is a primitive feature of the *Nematostella* Hox-related genes, while differential expression along the secondary body axis evolved afterwards.

Collectively, these data suggest that at least a rudimentary Hox code was operative in the cnidarian-bilaterian ancestor and that it played a role in patterning the animal's primary body axis (and possibly the secondary body axis as well). Moreover, strong stabilizing selection has been operating on this Hox code that has maintained certain core characteristics despite being deployed in a bewildering array of animal forms for over half a billion years.

## Results

### Phylogenetic Analysis

**Figure 1:**
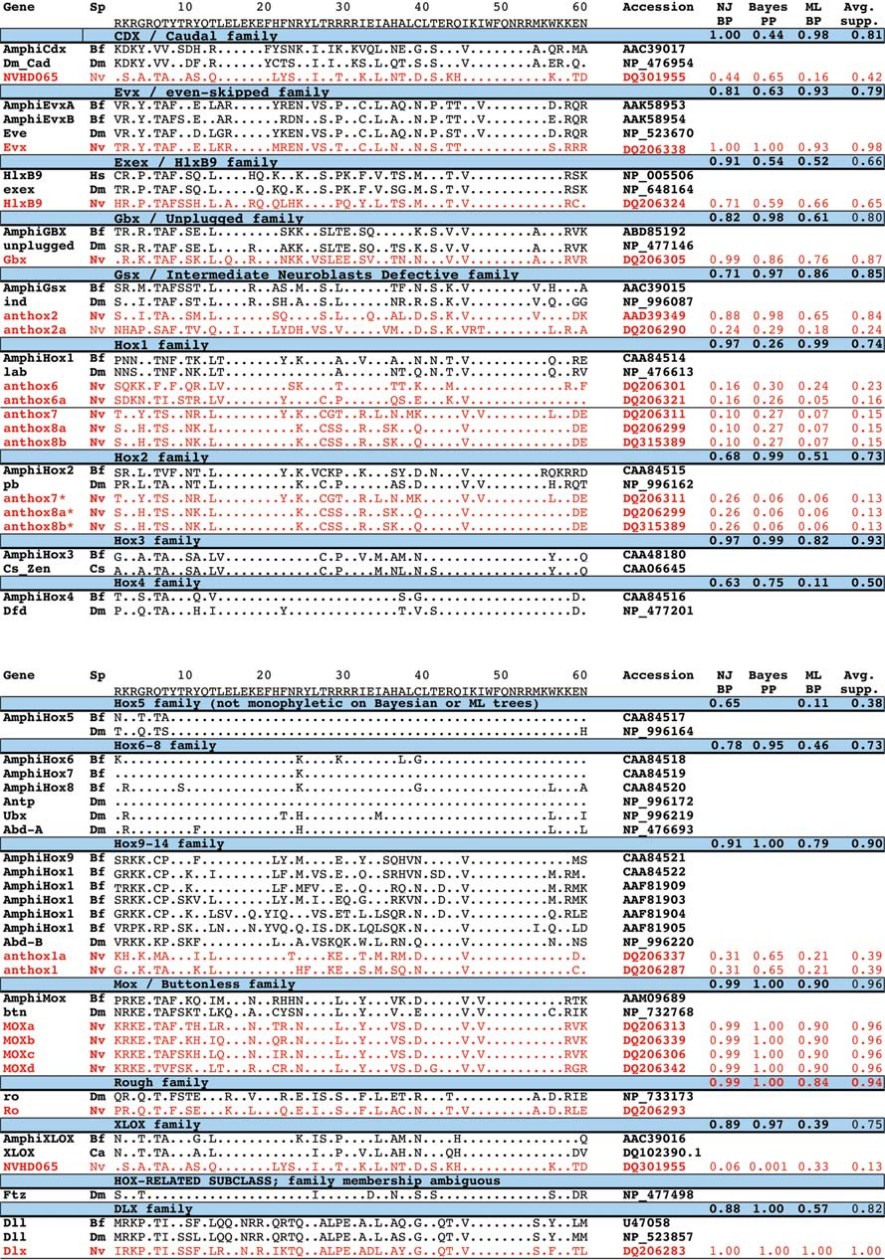
Alignment of homeodomains included in the phylogenetic analyses. All sequences are aligned to the *Drosophila* Antennapedia homeodomain. Each *Nematostella* sequence is grouped with putative bilaterian homologs. The degree of statistical support (bootstrap proporation [BP] or posterior probability [PP]) for each of these homology assignments is indicated separately for the neighbor-joining (NJ), Bayesian (Bayes), and maximum-likelihood (ML) trees. The average statistical support for each grouping is indicated in the far right column (NJ-BP+Bayes-PP+ML-BP/3). The dataset is available in Phylip format as Figure S12.

**Figure 2:**
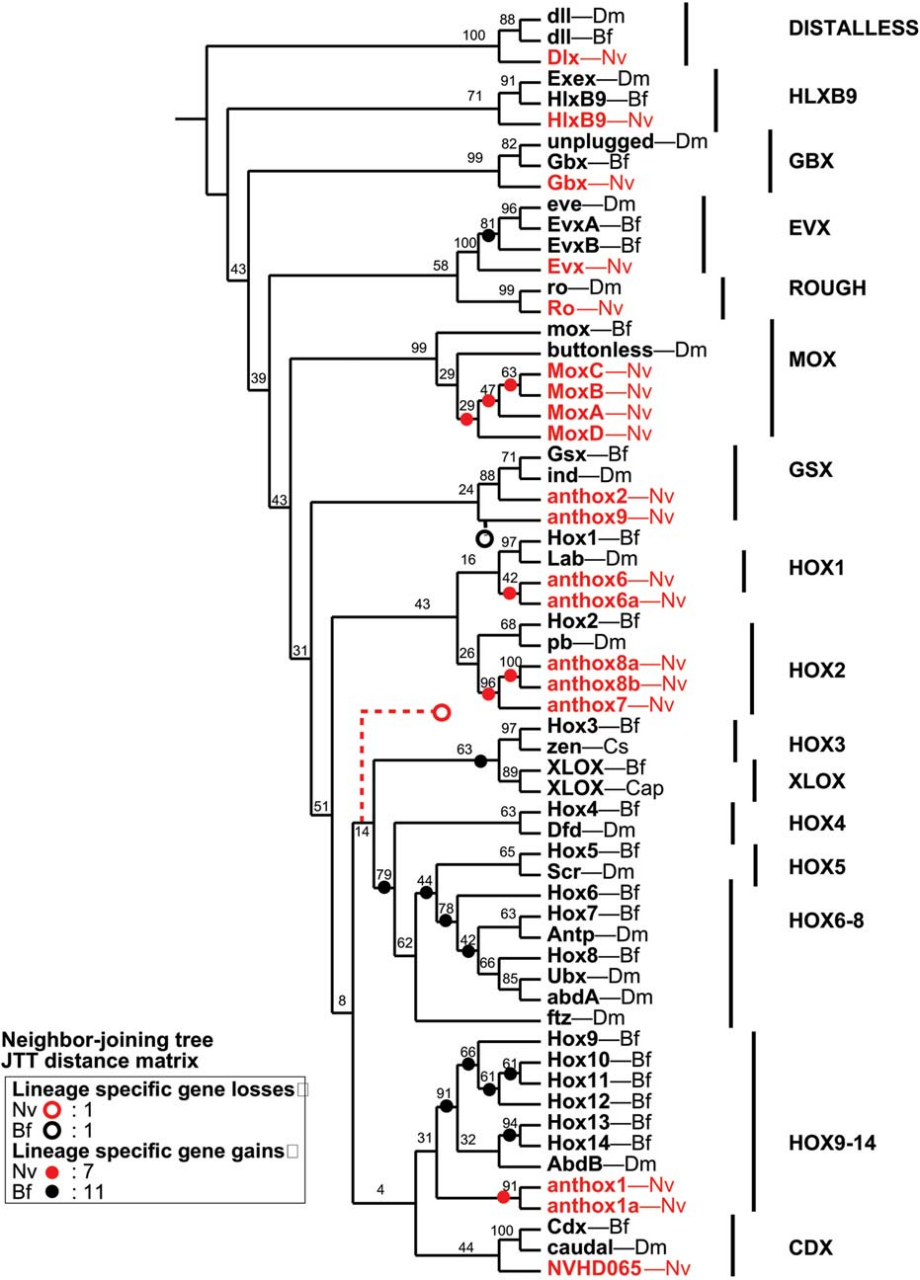
Homeodomain phylogeny based on neighbor-joining. The cladogram is rooted using the dll sequences. *Nematostella* sequences are shown in red. Bilaterian sequences are shown in black. Bootstrap proportions are presented at each node. Open circles depict implied gene losses for *Nematostella* (red) and *Branchiostoma* (black). Closed circles depict implied lineage-specific gene duplications for *Nematostella* (red) and *Branchiostoma* (black). The dataset used in this analysis is available as Figure S12.

Overall, there is substantial agreement among three different phylogenetic methods (neighbor-joining, maximum-likelihood, and Bayesian analysis) regarding the phylogenetic relationships of 18 Hox-related genes from the sea anemone *Nematostella* (phylum Cnidaria) and 43 Hox-related genes from representative Bilateria (Figure 1). On all three trees (Figure 2, S1, and S2), presumed protostome and deuterostome orthologs are grouped together with robust statistical support. In addition, all three trees indicate that the HlxB9, Gbx, Evx, and Rough families emerge basal to a clade that contains the Hox and ParaHox genes [50], [52]. On the neighbor-joining (Figure 2) and Bayesian trees (Figure S1), the Mox family also emerges basal to the Hox-ParaHox clade, but on the maximum-likelihood tree, Mox is nested within the Hox-ParaHox clade.

On all three trees, and in agreement with two recent studies, nine of *Nematostella's* sequences can be confidently assigned to the following six homeodomain families: HlxB9 (1), Gbx (1), Evx (1), Rough (1), Mox (4), Gsx (1) [50], [52]. The mean statistical support for these groupings, averaged over all three phylogenetic analyses, ranges from 0.65 to 1.00 (Figure 1).

Other groupings are also recovered on all three trees, but with somewhat lower statistical support. The anthox1 and anthox1a homeodomains cluster together on all three trees, suggesting that these two sequences were produced by a gene duplication that is specific to the Cnidaria. The anthox1/1a lineage appears most closely related to the posterior Hox genes (Hox9–14) on all three trees, with mean statistical support of 0.39. Anthox6 appears most closely related to the Hox1 family on all three trees, in agreement with previous studies [45], [46], [49], [52]. The mean statistical support for this grouping is 0.23 (Figure 1).

The precise placement of six *Nematostella* homeodomains varies between trees. Homeodomain NVHD065 groups with the Cdx family on the neighbor-joining and Bayesian trees. However, on the maximum-likelihood tree, NVHD065 groups with the Xlox family. The mean statistical support for the grouping of NVHD065 with Cdx (0.42) is higher than the mean statistical support for its grouping with Xlox (0.20). However, NVHD065 shares more identical amino acids with Xlox homeodomains (39/60 versus amphiXlox) than with Cdx homeodomains (30/60 versus amphiCdx). It also shares one distinctive residue with Xlox homeodomains (a histidine at position 44) and no distinctive residues with Cdx homeodomains. Chourrout and co-workers [51] have suggested that the NVHD065 gene (which they refer to as XLOX/CDX ) may be related to both XLOX and CDX.

Anthox6a [49] (known as NVHD060 in [52]) groups with anthox6 and appears most closely related to the Hox1 family on both the neighbor-joining and Bayesian trees (Figure 2; Figure S1). However, on the maximum-likelihood tree, anthox6a appears more closely related to the Gsx family (Figure S2). However, bootstrap support for the grouping of anthox6a with Gsx is very low; the bootstrap proportion (BP) is only 0.04. The mean statistical support (MS) for the node uniting anthox6, anthox6a, and Hox1 is substantially higher (MS = 0.16), suggesting that anthox6a could be a quickly evolving paralog of anthox6.

Anthox9 [49] (known as NVHD117 in [52]) appears most closely related to Gsx on the neighbor-joining and Bayesian trees (MS = 0.24). However, on the maximum-likelihood tree, this sequence emerges as the sister group to the Mox clade, albeit with very low bootstrap support (BP = 0.02). Kamm et. al. [49] point out that this predicted protein is peculiar in possessing an isoleucine residue at position 16 of the homeodomain, and they suggested that it may be a pseudogene. In support of this hypothesis, anthox9 is the only one of 20 Hox-related genes in the *Nematostella* genome for which we have failed to detect expression by *in situ* hybridization. On all three trees, anthox7, anthox8a, and anthox8b form a well-supported clade exclusive of any bilaterian sequences, which suggests that these three genes arose via two gene duplications within the Cnidaria. The precise placement of this anthox7/8a/8b lineage varies among all three trees. On the neighbor-joining tree (Figure 2), anthox7, 8a, and 8b group with the Hox2 family, a finding that is consistent with previous studies [45], [46]. However, on the Bayesian tree, anthox7/8a/8b appears more closely related to the Hox1 family. On the maximum-likelihood tree, anthox7/8a/8b emerges as an independent lineage, immediately after the emergence of a bilaterian Hox2 lineage.

### Structure and genomic arrangement of Hox and Hox-related genes

**Figure 3:**
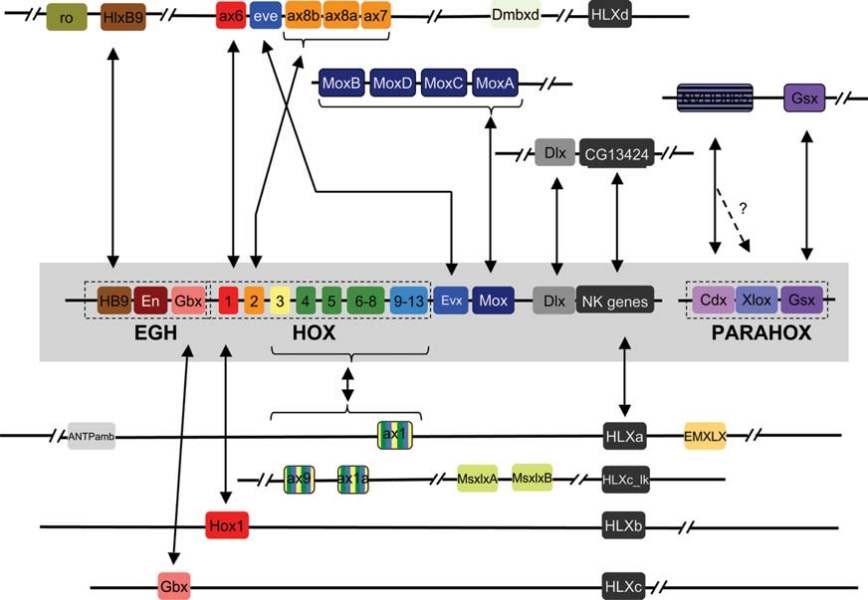
Clusters of Hox-related homeobox genes in the *Nematostella* genome. Based on the current genomic assemblies, thirty ANTP class genes of *Nematostella* are distributed among seven homeobox clusters [52]. The location of the PRD class gene Dmbx is also shown. The arrangement of the *Nematostella* genes is shown in relation to the hypothesized "extended Hox cluster," that is presumed to have existed in the most recent common ancestor of protostomes and deuterostomes (gray box; [127], [128]). Horizontal lines connecting *Nematostella* genes indicate known genomic linkage. Double-arrows connect *Nematostella* homeodomains to their putative bilaterian homologs based on phylogenetic analyses of homeodomain sequences (Figure 1; supp figs. 1–2). Detailed diagrams of each of the eight *Nematostella* homeodomain clusters are presented in supplemental figures 3 [Supplementary-material pone.0000153.s004] [Supplementary-material pone.0000153.s005] [Supplementary-material pone.0000153.s006] [Supplementary-material pone.0000153.s007] [Supplementary-material pone.0000153.s008] [Supplementary-material pone.0000153.s009]–10.

Based on two publicly available genome assemblies [53]–[55] and an abundance of corroborating spot-sequencing, the eighteen Hox and Hox-related genes of *Nematostella* are distributed among seven different genomic clusters (Figure 3). This finding is consistent with a recent report by Chourrout and co-workers [51]. Overall, the agreement between our spot sequencing and the genome traces is extremely high. For example, an anthox2 cDNA is a precise match for JGI scaffold 27 over its entire length of 1,019 nucleotides [46], and a 3,794 nucleotide stretch of the anthox1a locus that we isolated by ligation-mediated PCR is a 99.9% match to JGI scaffold 3, differing by four single nucleotide insertions.

The largest cluster comprises seven Hox-related genes, as follows: Rough—HlxB9—anthox6—Evx—anthox8b—anthox8a—anthox7 (Figure 3; Figure S3). The intergenic distances among these genes ranges from 2,868 nucleotides between anthox8a and anthox8b to 21,487 nucleotides between anthox8b and eve (Figure S3). With the exception of HlxB9, all of these genes exhibit the same transcriptional orientation. Except for Evx, which consists of three exons, these genes all contain two exons. In no case is the homeodomain interrupted by an intron. The PRD class homeobox gene Dmbx [52] is located more than 98,000 nucleotides upstream of anthox7, and the NK-related homeodomain HLXd is located more than 415,000 nucleotides upstream of Dmbx. There are no other homeodomains within 42,385 nucleotides downstream of Rough. Based on BLASTX searches against the NCBI Reference Sequence (RefSeq) database [56], a single non-homeobox gene resembling a hypothetical mouse protein (accession number XP_001000509.1; E-value = 9×10^−6^) is interposed between anthox8b and eve, while a sequence similar to a predicted *Staphylococcus* gene protein (accession number XP_251955; E-value = 2×10^−20^) is interposed between eve and anthox6 (Figure S3). Twenty non-homeobox genes are predicted to lie within the nearly 300 kilobases that separates anthox6 from HlxB9.

All four Mox genes reside in a compact genomic cluster, each having the same transcriptional orientation (Figure S4). From the N-terminal exon of MoxB to the C-terminal exon of MoxA, the cluster spans 23,210 nucleotides. No other homeobox genes reside within 48 kilobases upstream or 40 kilobases downstream of the Mox cluster. Roughly nine kilobases upstream of the Mox cluster, BLASTX identifies a protein with significant similarity to the PF20 protein of *Chlamydomonas* (accession number T08180; E-value = 8×10^−24^). Roughly 5 kilobases downstream of the Mox cluster, BLASTX identifies a sequence with significant similarity to a retrotransposon (accession number BAD86655.1; E-value 2×10^−27^). The BLASTX search returned no significant hits within the intergenic regions (cutoff E-value<0.0001).

The two putative paraHox genes (anthox2 and NVHD065) are closely linked on a single genomic scaffold in the Phusion assembly (Figure 3; Figure S5). Anthox2, a clear Gsx ortholog [46], is separated by 12,977 nucleotides from NVHD065, a sequence that appears most closely related to Cdx on the neighbor-joining and Bayesian trees (Figure 2; Figure S1) but most closely related to Xlox on the maximum-likelihood tree (Figure S2). The two genes exhibit the same transcriptional orientation. Both genes possess two exons, with the homeodomain encoded entirely by the second exon. Anthox2 has a short intron (176 nucleotides), while NVHD065 has a long intron (10,529 nucleotides). A 1,580,677 nucleotide scaffold encompassing this two-gene cluster was recovered from the JGI assembly of the *Nematostella* genome. A predicted gene with apparent homology to the largest subunit of RNA Polymerase II (accession number XP_001056421.1) is located approximately 5,500 nucleotides upstream of Gsx, while a gene with apparent homology to a predicted DNA-polymerase (accession number XP_785333.1) is located approximately 7,000 nucleotides downstream of NVHD065.

Anthox9 [51], is very tightly linked to the posterior Hox gene anthox1a (Figure S6). The two genes exhibit the opposite orientation. There are no other genes predicted to occur in the 3,865-nucleotide intergenic region. Anthox1, anthox6a, and Gbx do not appear closely linked to other Hox-related genes, although all three genes are distantly linked to homeobox genes from the HLX family. Anthox1 is over 300,000 nucleotides distant from HLXa (Figure S7). Anthox6a is over 263,000 nucleotides distant from HLXb (Figure S8). Gbx is over 600,000 nucleotides distant from HLXc (Figure S9). Interestingly, different HLX genes are also distantly linked to the anterior Hox cluster (HLXd; Figure S3) and the anthox1a/anthox9 cluster (HLXc-like; Figure S6). Finally, the Dlx gene is only 6,988 nucleotides distant from NVHD021 (Figure S10). NVHD021 (accession number DQ206308) is an ortholog to the *Drosophila* ANTP class gene, CG13424, and is more distantly related to the Hox genes than most other genes included in this study [50], [52].

### Gene Expression of basal Hox-related genes in Nematostella

**Figure 4:**
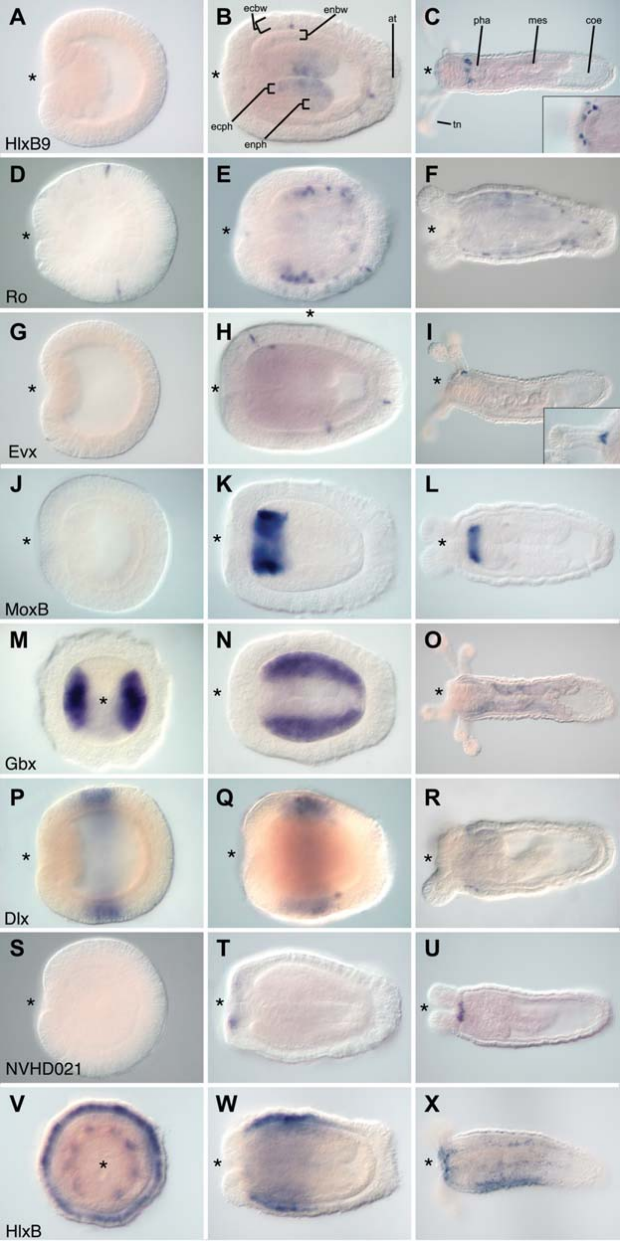
Developmental Expression of Hox-ParaHox related genes in *Nematostella*. Gene expression was assayed throughout embryonic and larval development using *in situ* hybridization. All images are optical sections that permit visualization of the endodermal tissue layer. Panels M and V are transverse sections, but all other images are longitudinal sections with the future oral end of the animal facing left. The blastopore (site of the future mouth) is indicated by an asterisk. Abbreviations are as follows: apical tuft (at); coelenterone (coe); bodywall ectoderm (ecbw); pharyngeal ectoderm (ecph); bodywall endoderm (enbw); pharyngeal endoderm (enph); mesentery (mes); pharynx (pha); tentacle (tn).

**Figure 5:**
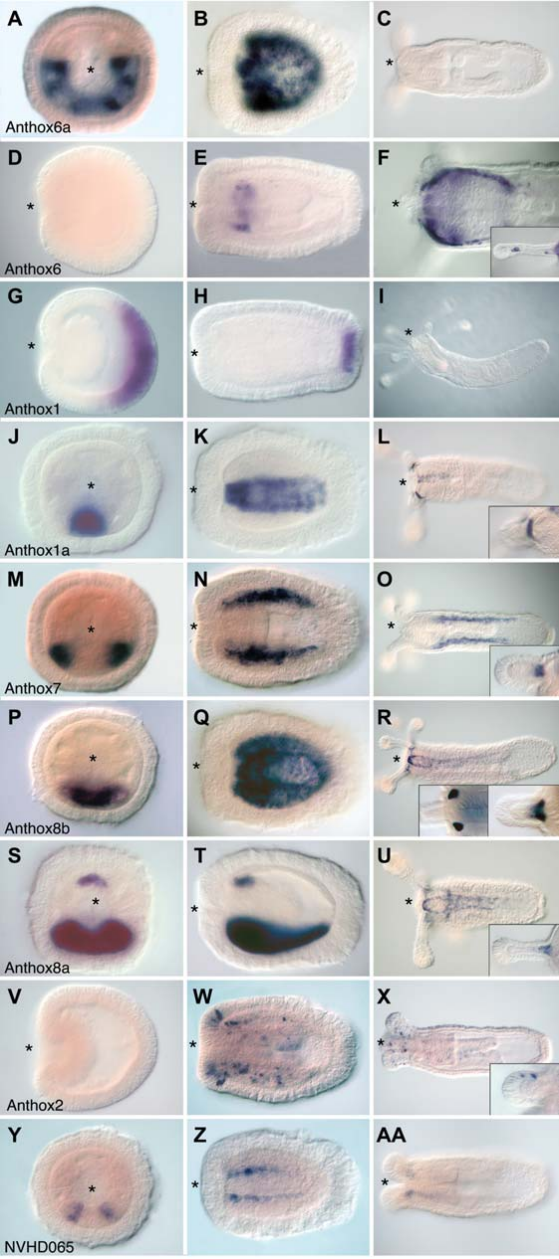
Developmental Expression of Hox and ParaHox homologs in *Nematostella*. Gene expression was assayed throughout embryonic and larval development using *in situ* hybridization. All images are optical sections that permit visualization of the endodermal tissue layer. Panels A, J, M, P, S, and Y are transverse sections, but all other images are longitudinal sections with the future oral end of the animal facing left. The blastopore, which becomes the mouth, is indicated by an asterisk. Abbreviations are as follows: apical tuft (at); coelenterone (coe); bodywall ectoderm (ecbw); pharyngeal ectoderm (ecph); bodywall endoderm (enbw); pharyngeal endoderm (enph); mesentery (mes); pharynx (pha); tentacle (tn).

The developmental expression of 20 ANTP class homeobox genes is reported here (Figure 4 and 5), including seven putative Hox genes (anthox1, anthox1a, anthox6, anthox6a, anthox7, anthox8a, and anthox8b), two putative ParaHox genes (anthox2 and NVHD065), eight basal Hox-related genes (HlxB9, Gbx, Rough, Evx, and four Mox genes) and three more distantly related ANTP homeobox genes (Dlx, HLXb, and a NVHD021). Expression of another Hox-related gene (anthox9) could not be detected. The expression of 13 of these genes has not previously been described. For seven of these genes, we present new views or new developmental stages and describe novel aspects of their developmental expression.

HlxB9 transcripts are not detected until after gastrulation (Figure 4A–C). At planula stages, the majority of expression is in cells of the pharyngeal ectoderm. In addition, a small number of cells scattered throughout the body wall ectoderm are also expressing HlxB9. By polyp stages, HlxB9 expressing cells are concentrated in a ring around the pharynx (Figure 4C) and in the ectoderm between the tentacles (Figure 4C, inset).

Rough is first detected by *in situ* hybridization during mid-gastrulation in cells that span the ectodermal epithelium (Figure 4D). In the planula larva and the juvenile polyp, transcripts can be seen in both ectodermal and body wall endoderm in the center of the body column and in the aboral region (Figure 4E and 4F). Rough-expressing cells are not seen at the oral pole or in the developing tentacles. The basal location of the mature cell bodies suggests that these cells may be a subset of neurons.

Evx expression first becomes apparent in the planula larva, in scattered columnar cells located along the basal surface of the ectodermal epithelium, where the outer ectodermal layer meets the mesoglea (Figure 4H). No Evx expression is observed at the oral or aboral extremities, *i.e.*, around the mouth or in the foot. The identity of the Evx-expressing cells is not known with certainty, but their morphology and location are consistent with them being a subset of sensory neurons (Meg Daly, personal communication). In the juvenile polyp, Evx-expressing cells appear exclusively in the endoderm at the base of the tentacles (Figure 4I, inset).

The expression patterns of the four Mox genes are indistinguishable. MoxB expression is depicted in Figures 4J–4L. As with Evx, expression is not evident prior to gastrulation (data not shown). Following gastrulation, Mox is expressed in a ring of endoderm in the pharyngeal region of the planula. Expression persists in this ring of pharyngeal endoderm after the tentacles have emerged and the planula has begun to assume the form of the adult polyp (Figure 4L). Mox is not expressed in the body wall endoderm or the ectoderm. Expression of the other three Mox genes is illustrated in Figure S11.

Gbx expression has been described previously [57]. Expression begins during early planula stages on the left and right sides of the directive axis in body wall endoderm (Figure 4M and 4N). During later stages, expression is down regulated in body wall endoderm and initiates, and persists in pharyngeal endoderm on the left and right sides (Figure 4O).

### Gene Expression of ANTP genes more distantly related to Hox in Nematostella

Dlx expression first becomes apparent during the later stages of gastrulation, as a circumferential ring of ectodermal cells in the center of the body column (Figure 4P). Expression persists throughout planula stages but continues to be excluded from the oral and aboral poles (Figure 4Q). In the juvenile polyp, a layer of Dlx expressing cells resides in a basal position within the ectodermal epithelium, at the level of the pharynx (Figure 4R). Some or all of the Dlx expressing cells may be neural precursors based on their initial and final basal nuclear position in the ectodermal epithelium.

NVHD021 expression first becomes apparent in the oral ectodermal region of the planula larva (Figure 4T). Its expression persists in a more concentrated ring around the mouth of the juvenile polyp (Figure 4U).

HLXb transcripts accumulate during late planula stages in body wall ectoderm overlying the pharyngeal region (capitulum), and in eight domains of pharyngeal endoderm that correspond to the eight prospective mesenteries (Figure 4V and 4W). The mesenteries will eventually grow out to meet mesenterial precursors in the body wall endoderm. Expression in both of these regions persists throughout polyp stages and is upregulated in oral tissues in the juvenile polyp (Figure 4X). HLXb (accession number DQ206303) is an ortholog to the Human ANTP class gene, HLX1, and is more distantly related to the Hox genes than most other genes included in this study [52].

### Expression of Hox and ParaHox genes in Nematostella

The terms dorsal and ventral have been applied to the directive axis of cnidarians, specifically as an aid to naming mesenteries [58]. However, the use of these terms does not imply definitive homology between the directive axis of cnidarians and the dorsal-ventral axis of bilaterians. It has recently been shown that many genes responsible for dorsoventral patterning in Bilateria are also expressed asymmetrically about the directive axis in Cnidaria [36], [57], [59]–[61]. However, because the expression patterns of dorsoventral patterning genes are not entirely consistent between Cnidaria and Bilateria, the molecular data do not make clear whether one side of the directive axis corresponds to the dorsal of Bilateria, and the other side to ventral. Here, we will adopt the use of these terms to describe gene expression patterns and designate specific mesenteries without implying homology with the “dorsal” and “ventral” of Bilateria. We will refer to the side opposite the siphonoglyph [58], a ciliated groove in the pharynx of *Nematostella*, as ventral.

Anthox6a is expressed in the bodywall endoderm and the presumptive mesenteries of the early planula (Figure 5A and 5B), but it is not expressed in the juvenile polyp (Figure 5C). A transverse section reveals that the expression is restricted along the secondary or directive axis; about 2/3 of the endodermal tissue expresses anthox6a. Six out of the eight presumptive mesenteries express anthox6a, and the sharp expression boundary appears to coincide with one pair of mesenteries. Double *in situ* labeling reveals that anthox6a is expressed on the ventral side of the directive axis (data not shown).

**Figure 6:**
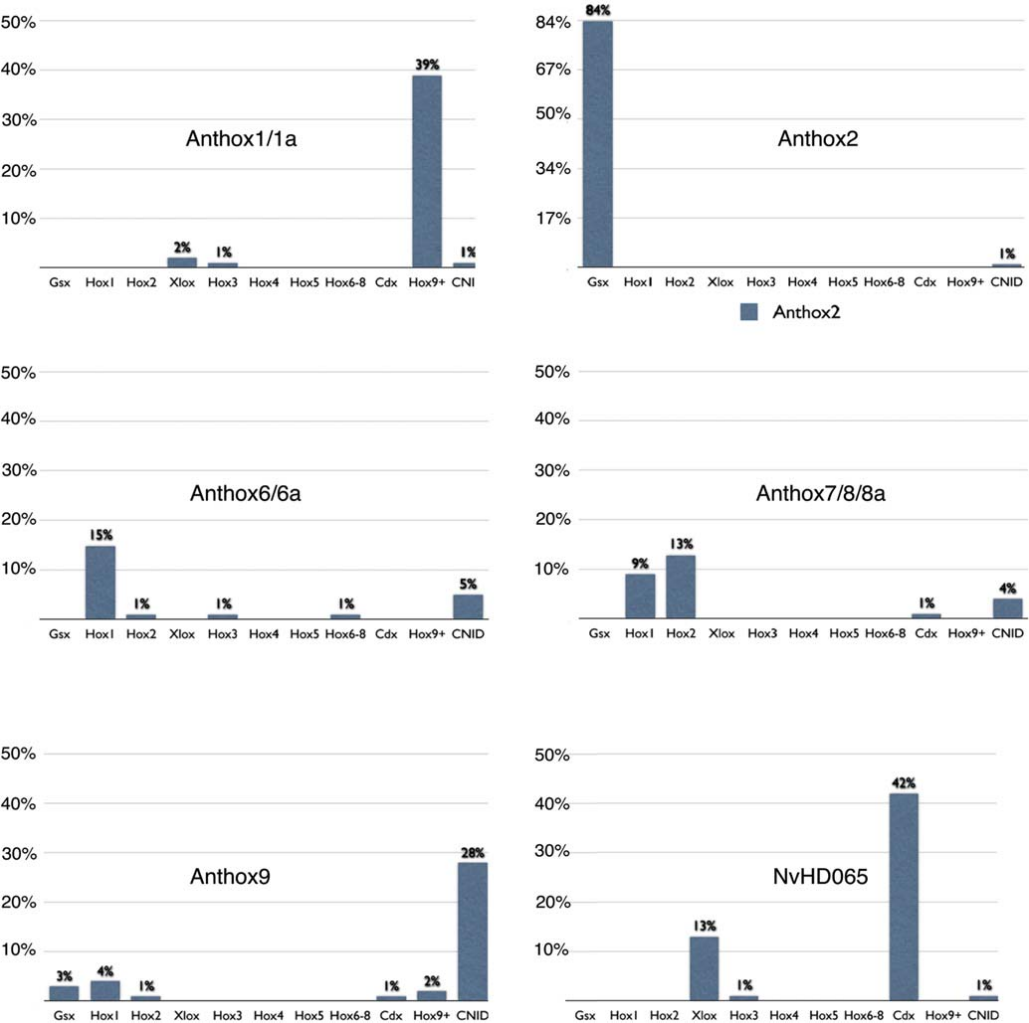
Mean statistical support for select phylogenetic groupings. The mean statistical support for hypothetical clades linking each of six *Nematostella* Hox/ParaHox lineages with potential homologs in the Bilateria is depicted graphically. The mean statistical support is the average of the neighbor-joining bootstrap proportion, the Bayesian posterior probability, and the maximum-likelihood bootstrap proportion. It is expressed as a percent of trees in which the given grouping was recovered. Individual *Nematostella* genes were grouped into lineages (for example, anthox1-anthox1a) when the mean statistical support for the clade uniting them exceeded the mean statistical support for any other competing relationship. The final column depicts the highest support obtained for any pairwise relationship with another cnidarian homeodomain.

The early expression of anthox6 has been described previously [36]. Expression first becomes visible during early planula stages as a ring in the pharyngeal endoderm (Figure 5E). This pattern persists in the juvenile polyp, and it extends orally to the mouth opening (Figure 5D). The region expressing anthox6 comes to include the endoderm at the base of the tentacles and scattered cells at the tips of the elongating tentacles (Figure 5D, inset).

Anthox1 expression begins at late blastula stages, in the vegetal hemisphere of the embryo and earlier than any other Hox gene in *Nematostella*; expression then persists through gastrulation (Figure 5G and 5H). In the planula, anthox1 expression persists and becomes refined to the apical tuft of sensory cilia at the leading swimming end of the planula (aboral end of the adult polyp). Expression wanes in the polyp (Figure 5I). This putative posterior Hox gene is expressed at the opposite side of the oral-aboral axis from the anterior Hox gene anthox6, as described previously [36].

Anthox1a is the sister gene of the putative posterior Hox gene anthox1, but its expression is more similar to putative anterior Hox genes, such as anthox6a. Like anthox6a, anthox1a exhibits extremely restricted expression along the directive axis (Figure 5J–K). This gene is expressed in a thin stripe of body wall endoderm flanking the ventral midline. This region gives rise to the ventral surfaces of the ventral pair of mesenteries in the adult. Expression persists in these two mesenteries in the juvenile polyp stage (Figure 5L). Expression is also seen in the endoderm at base of the tentacles (Figure 5L, inset).

Anthox7, anthox8a, and anthox8b are expressed in the body wall endoderm, in nested domains centered about the ventral midline (Figure 5M–5U). The expression of these genes first becomes visible in the early larva, and it persists into the juvenile polyp stage. Anthox7 is expressed in a pair of narrow, bilaterally symmetrical bands of body-wall endoderm (Figure 5M). Anthox8a is expressed in a single broad band along the ventral midline that encompasses the expression territories of both anthox1a and anthox7. Anthox8a is expressed along the ventral midline in the same basic expression territory as its sister gene anthox8b (encompassing the expression territories of both anthox7 and anthox1a) (Figure 5P–5U). However, anthox8a also exhibits an arc of expression in the pharyngeal endoderm along the dorsal surface of the pharynx. This expression corresponds to a subset of the pharyngeal ring of expression exhibited by the anterior Hox gene anthox6 (Figure 5S and 5T). Anthox8a and anthox8b continue to be expressed in the ventral pair of mesenteries in the juvenile polyp (Figure 5R and 5U), while anthox7 is expressed in the adjacent pair of ventral-lateral mesenteries (Figure 5O). Both anthox8a and anthox8b are expressed in the endoderm at the base of the tentacles, with anthox8a being expressed earlier, before tentacle outgrowth is initiated. The expression of anthox7 and anthox8a, but not anthox8b, have been described previously [36], [59].

Anthox2 is a Gsx (anterior ParaHox) ortholog [46] whose expression has been described elsewhere [62]. Transcripts accumulate in scattered cells around the oral pole in the ectoderm of the body column, the pharynx, and the tentacles (Figure 5V–5X). These cells appear to be neural precursors based on cell morphology. No aboral expression is observed. Anthox2 does not exhibit any asymmetry about the directive axis.

NVHD065 is expressed during early planula stages in two thin stripes along the ventral midline (Figure 5Y–5Z). These two stripes persist into the polyp stage (Figure 5AA). The cells expressing NVHD065 are located in the same region as the cells that express anthox1a, anthox8a, and anthox8b. These cells contribute to the development of the ventral pair of mesenteries.

## Discussion

### Do cnidarians possess “true” Hox genes?

Numerous Hox-related genes have been recovered from several cnidarian model systems over the last fifteen years [44], [46], [49], [50], [63]–[70], but the precise identity of these genes has been a contentious issue. Some of the earliest homeobox sequences recovered from the phylum Cnidaria were initially identified as anterior and central Hox genes (lab/pb-like and deformed-like) [69]–[74]. Murtha and co-workers identified two homeobox fragments from *Sarsia* as likely Hox1 (lab) and Hox2 (pb) homologs [70]. In addition, they suggested that a primitive axial patterning system featuring Hox genes could have evolved prior to the evolutionary split between Cnidaria and Bilateria; they also suggested that the anterior Hox genes might have evolved before the more posterior Hox genes [70]. Both of these hypotheses are consistent with the findings of the present study. However, the orthology assignments made in these early studies were of limited reliability since they were based on pairwise alignments [69], [70], [72]–[74] or, in one case, on a phylogenetic analysis that encompassed too narrow a sample of bilaterian homeobox genes [71].

Subsequent studies relied increasingly on phylogenetic analysis of homeodomain sequences for orthology assignments. These studies consistently reported the existence of anterior (hox1 and/or hox2 related) and posterior (hox9–14 related) homeodomains in the Cnidaria [44], [46], [48], [63], [64], [75]. However, clear homologs of the other bilaterian Hox families (hox3–hox8) were not identified. In addition, a clear ortholog of the bilaterian Gsx gene, an “anterior” ParaHox gene was identified in the Cnidaria [46]. At the same time, a less convincing possible ortholog of Cdx, a “posterior” ParaHox gene was identified [46]. The discovery of ParaHox genes in Cnidaria is important given that Hox genes, by themselves, do not constitute a monophyletic group. The most recent common ancestor of the Hox genes appears to have given rise also to some or all of the ParaHox genes (Gsx, Xlox, and Cdx) [76], although the exact nature of the relationship of the Hox and ParaHox genes is still unclear [41], [47], [51]. Regardless of their exact relationship, the evolution of Hox genes cannot be considered in isolation from that of the ParaHox genes.

With the sequencing of the complete genome of the sea anemone *Nematostella vectensis*, the Hox complement of cnidarians is being re-evaluated. Kamm and co-workers [49] culled nine Hox-related homeodomains from the genome of *Nematostella* (six of which had been previously described), and compared these, along with four Hox-related genes from the colonial hydrozoan *Eleutheria*, to bilaterian Hox and ParaHox genes using phylogenetic analysis. (The Kamm et al. 2006 study did not include the NVHD065 homeodomain included in the current study [49].) In agreement with numerous previous studies [44], [46], [48], [63], [64], [75], the Kamm et al. phylogenetic analyses [49] supported the existence of a cnidarian Gsx gene, the existence of a cnidarian Hox1 gene, the existence of a cnidarian Cdx gene, and the absence of central Hox genes (Hox3–8) in Cnidaria. However, Kamm and co-workers [49] differ from previous studies in concluding that cnidarians lack any other “true” Hox genes, aside from Hox1.

More recently, Chourrout and co-workers came to a similar conclusion; they contend that anterior Hox genes likely predated the cnidarian-bilaterian split, but that “non-anterior genes could have appeared independently in the Hox and ParaHox clusters, possibly after the separation of bilaterians and cnidarians” [51]. This conclusion was largely based on an unorthodox phylogenetic treatment of cnidarian and bilaterian homeodomain sequences that employed Neighbor-net analysis. Neighbor-net analysis is based on the neighbor-joining method [77], but it is explicitly designed to investigate and visualize “complex evolutionary scenarios” that cannot be accurately modeled by a bifurcating phylogenetic tree, *for example*, scenarios involving reticulate evolution such as gene recombination, hybridization, and horizontal gene transfer [78], [79]. The authors do not explicitly justify their use of Neighbor-net analysis rather than more traditional methods that produce bifurcating trees. The Hox radiation is thought to have occurred via a bifurcating process that involved repeated rounds of gene duplication and divergence. In support of this, there are many well-supported nodes in published Hox phylogenies. The Neighbour-network diagram presented by Chourrout et al. can make it more difficult to visualize these well-supported nodes because it is a two dimensional projection of a three-dimensional graph. We suggest that Neighbour-net analysis would have been more appropriate as a supplement to rather than a replacement for a traditional tree-building algorithm.

Below, we argue that recent studies systematically underestimate the phylogenetic support for the existence of multiple Hox genes in the cnidarian-bilaterian ancestor due to three important logical shortcomings. (1) These studies do not explicitly consider the weakness of the statistical support for the competing phylogenetic hypotheses that are implied or explicitly stated; (2) they do not account for the number of independent gene gains and losses that would be required by competing phylogenetic hypotheses; and (3) they do not properly root the Hox-ParaHox radiation with a succession of closely related outgroup genes (*for example*, HlxB9, Gbx, Evx, Rough, and Mox). When these issues are addressed, it becomes apparent that the support for three or more “true” Hox genes in the cnidarian-bilaterian ancestor is considerably greater than the support for only two [51], one [49], [51], or even none [50].

### Statistical support for competing hypotheses

In absolute terms, the statistical support for the nodes uniting cnidarian and bilaterian Hox and ParaHox genes is generally modest. The reasons for this modest statistical support include the large number of taxa involved, the small number of phylogenetic characters, the occurrence of lineage specific Hox gene duplications, and the (likely) short evolutionary interval between the initial Hox-ParaHox radiation and the cnidarian-bilaterian divergence. However, the support for these critical nodes is substantially higher than the support for the hypothetical nodes that are predicted by competing hypotheses (Figure 6). For example, while the mean statistical support for the clade linking anthox1/1a with Hox9+ is modest (0.39), the mean statistical support for any other pairing is practically non-existent: the pairing of anthox1/1a with Cdx receives a mean statistical support of 0.04, the pairing with Hox3/zen receives a mean statistical support of 0.02, and the pairing with Xlox receives a mean statistical support of 0.01. The mean statistical support for all other pairwise groupings between anthox1/1a and bilaterian families was 0.00. Furthermore, the highest support for a clade linking anthox1/1a to another *Nematostella* lineage was only 0.01. Clearly, the best-supported relationship for anthox1/1a is to the Hox9+ family. A similar approach bolsters the support for a close relationship between anthox2 and Gsx, between anthox6 and Hox1, and between NvHD065 and Cdx. The only case where competing pairings receive comparable support is anthox7/8/8a. The mean statistical support grouping this *Nematostella* lineage with the Hox2 family (0.13) is only slightly higher than the mean statistical support for grouping it with the Hox1 family (0.09). However, the analysis provides no support for the hypothesis that anthox7/8/8a is orthologous to Hox3, as recently proposed [51]—the mean statistical support for this grouping is 0.00.

**Figure 7:**
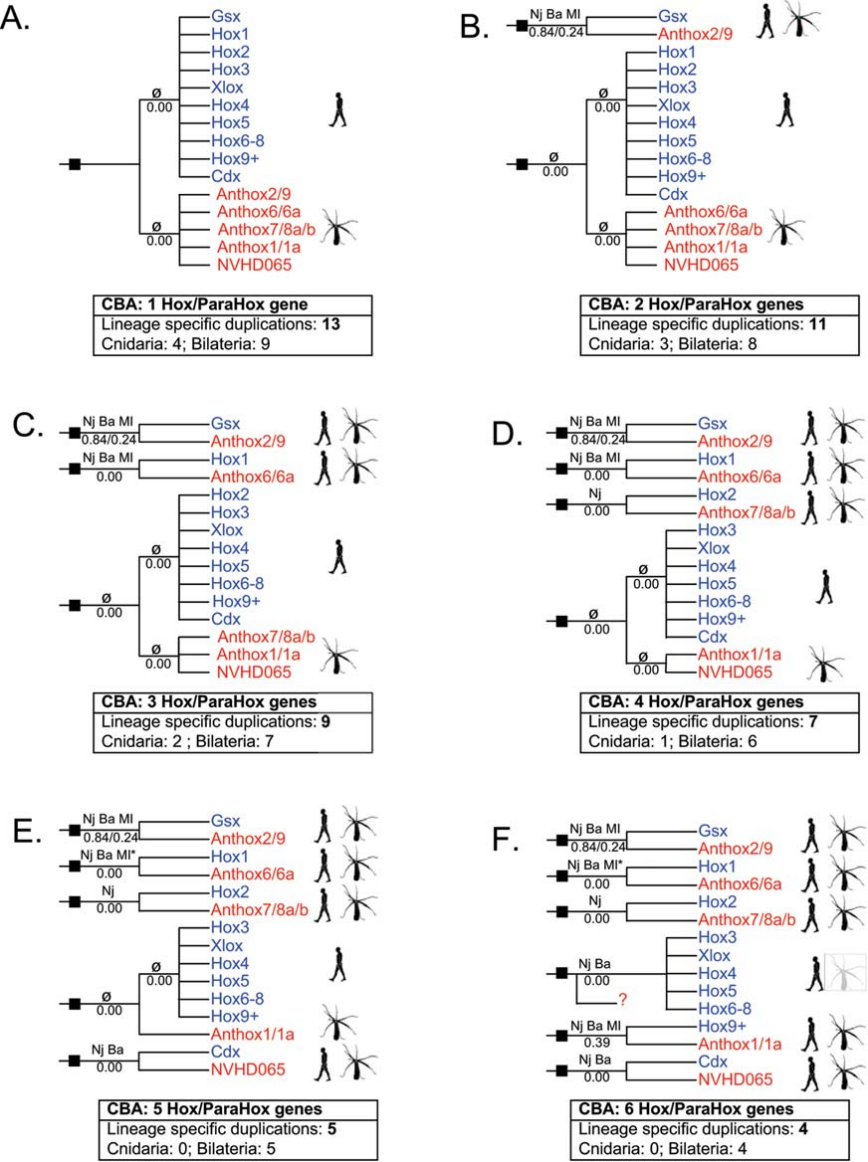
Hox/ParaHox evolutionary scenarios. The phylogenies drawn here depict six mutually exclusive scenarios regarding the evolution of the Hox and ParaHox genes. Ten distinct Hox and ParaHox lineages are thought to have been present in the ancestral bilaterian (Hox1, Hox2, Hox3, Hox4, Hox5, Hox6–8, Hox9+, Cdx, Gsx, and Xlox). Five distinct Hox/ParaHox lineages are recognized for *Nematostella*. (*Nematostella* homeodomains that tend to cluster together in the phylogenetic analyses are grouped together here: anthox1/1a; anthox2/9; anthox6/6a; anthox7/8a/8b.) Assuming no gene loss in the Cnidaria, then the existence of five Hox/ParaHox lineages in *Nematostella* implies that the cnidarian-bilaterian ancestor (CBA) could have possessed as few as one Hox/ParaHox gene (scenario A) or as many as five (scenario E). There is some indication that a central class Hox gene was lost in the Cnidaria [47], and that the CBA may have possessed six distinct Hox/ParaHox genes (scenario F). The ancestral Hox/ParaHox genes present in the CBA are indicated by solid squares. If a particular hypothetical clade is recovered on one or more of the phylogenetic analyses presented here, this is indicated above the relevant branch (NJ = neighbor-joining, Figure 2; Ba = Bayesian inference, Figure S1; ML = maximum- likelihood, Figure S2; φ = none). Below each branch, the average statistical support is indicated (NJ-bootstrap proportion+Bayes-posterior probability+ML-boostrap proportion/3). Each scenario implies a different number of lineage-specific gene losses.

Figure 7 compares the statistical support for larger phylogenetic groupings that are predicted by competing evolutionary scenarios. For example, if the hypothesis that cnidarians lack “true” Hox genes recently put forth by Kamm and Schierwater [50] is strictly interpreted from a phylogenetic standpoint, this would imply that all of the bilaterian Hox genes share a most recent common ancestor to the exclusion of any cnidarian genes (as in Figure 7A–B). However, the mean statistical support for the clade that unites all bilaterian Hox genes (Hox1–Hox14) to the exclusion of all cnidarian genes is 0.00. Contrast this with the mean statistical support for the clade uniting anthox1/1a with Hox9+ (0.39), the clade uniting anthox6 with Hox1 (0.23), and the clade uniting anthox7/8a/8b with Hox2 (0.13). Viewed in this way, the phylogenetic analyses provide substantially greater support for the existence of five or six Hox/ParaHox genes in the cnidarian-bilaterian ancestor (Figure 7E–F) than for the existence of only one, two, or three Hox/ParaHox genes in the cnidarian-bilaterian ancestor (Figure 7A–C).

### Implied gene gains and losses

Nearly all homeodomain phylogenies that have been published recently are derived from alignments of amino acid sequences. Branch lengths and topologies are optimized in order to minimize the amount of amino acid substitution that is inferred. However, preferred tree topologies do not account for gene duplication or gene loss. Compared to amino acid substitutions, gene losses and gene gains are rare evolutionary events, and they ought to be considered when attempting to choose among alternate hypotheses. Given two competing topologies that require roughly equivalent amounts of amino acid evolution, the topology that requires fewer gene gains and losses is preferable because it presupposes fewer of these relatively rare and typically unsubstantiated evolutionary events.

Among the analyses presented here, the neighbor-joining tree requires fewer lineage-specific gene losses and gains (20) than either the Bayesian tree (25) or the maximum-likelihood tree (23). The Bayesian tree is particularly unparsimonious with respect to implied gene losses by *Nematostella* (Figure S1); it requires that *Nematostella* underwent secondary loss of Hox2, a Hox3/Xlox precursor, Hox4, Hox5, and a Hox6–8 precursor. The maximum-likelihood tree requires that *Nematostella* lost Hox2, Hox3, and a Hox4–8/Cdx precursor (Figure S2). By contrast, the neighbor-joining tree requires that *Nematostella* lost a single gene (a Hox3–Hox8/Xlox precursor).

**Figure 8:**
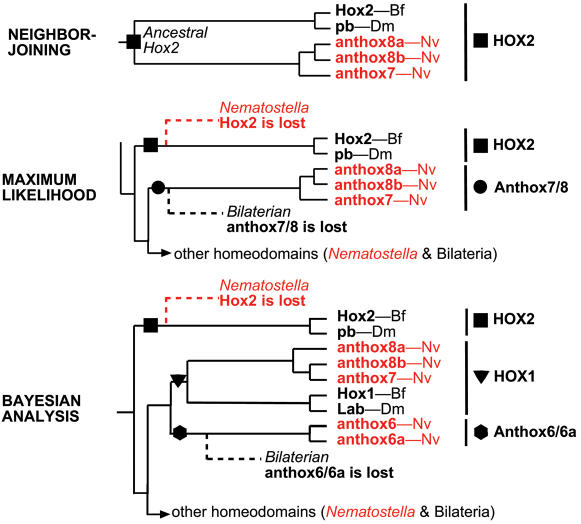
Reconstruction of Hox2 evolution. Portions of the neighbor-joining tree, the maximum-likelihood tree, and the Bayesian tree are redrawn here. The neighbor-joining tree implies that anthox7, 8a, and 8b are direct descendants of the ancestral Hox2 gene in the cnidarian-bilaterian ancestor. No gene loss is required. The maximum-likelihood tree implies that both a Hox2 precursor (square) and an anthox7/8a/8b precursor (circle) were present in the cnidarian-bilaterian ancestor. Hox2 was lost in the line leading to *Nematostella*, while anthox7/8a/8b ortholog was lost in the line leading to Bilateria. The Bayesian tree implies that a Hox2 precursor (square), a Hox1 parecursor (triangle), and an anthox6/6a precursor were present in the cnidarian-bilaterian ancestor. Hox2 was lost in the line leading to *Nematostella*, while anthox6/6a was lost in the line leading to Bilateria.

The issue of gene loss is particularly pertinent for reconstructing the evolution of the Hox2 family (Figure 8). On the neighbor-joining tree, anthox7/8a/8b is paired with Hox2/pb (Figure 8A). This topology suggests that Hox2 was present in the cnidarian-bilaterian ancestor. No gene loss is implied. However, on the maximum-likelihood tree, anthox7/8a/8b and Hox2/pb appear as independent gene lineages that emerged one after the other (Figure 8B). This arrangement implies two gene losses: the loss of a Hox2 gene in *Nematostella* and the loss of an anthox7/8 gene in the Bilateria. Finally, on the Bayesian tree, anthox7/8a/8b is grouped with Hox1/labial (Figure 8C). Anthox6/6a and Hox2/pb appear as successive outgroups to this putative Hox1 clade. This topology implies that three anterior Hox genes were present in the cnidarian-bilaterian ancestor: Hox1, Hox2, and an anthox6/6a precursor. Subsequently, the Hox2 gene was lost in the line leading to *Nematostella* and the anthox6/6a precursor was lost in the line leading to bilaterians. The scenario implied by the neighbor-joining tree is the most parsimonious, and based on this rationale, we favor the phylogenetic hypothesis generated by the neighbor-joining analysis and its implications that the cnidarian-bilaterian ancestor possessed a Hox2 gene.

### Absence of Central Hox Genes in the Phylum Cnidaria

Numerous studies have commented on the apparent absence of central Hox genes (Hox4–Hox8) in the phylum Cnidaria [44]–[46], [51], [52]. The lack of central genes in the Cnidaria could be explained (1) if the central Hox genes arose in the bilaterian lineage, or (2) if the central Hox genes originated prior to the cnidarian-bilaterian divergence, but they were subsequently lost in the Cnidaria. The phylogenetic analyses performed in this study tend to support the latter. On the neighbor-joining tree (Fig. 2), the central Hox genes appear as the sister group to a Hox3-Xlox clade. No *Nematostella* sequences fall within this clade. However, the tree implies that the common ancestor of this clade evolved prior to the cnidarian-bilaterian divergence, and that direct descendants of this ancestral gene were lost in the line leading to *Nematostella*. The maximum likelihood tree (Fig. S2) groups the central Hox genes with the Cdx genes. This tree likewise implies that a single gene was lost in the line leading to *Nematostella*. The Bayesian tree is least parsimonious with respect to the absence of central Hox genes in the Cnidaria (Fig. S1). The Bayesian tree implies that three central Hox genes were present in the cnidarian-bilaterian ancestor (Hox4, Hox5, and Hox6–8), and all three were lost in the lineage leading to *Nematostella*. Recently, Ryan and co-workers suggested a hypothesis that could explain the absence of central Hox genes in the Cnidaria without requiring gene loss in the Cnidaria. A Bayesian analysis of 455 homeodomains from human, fruit fly, and *Nematostella* placed anthox1 and anthox1a as the sister group to a clade comprising the central Hox genes and the posterior Hox genes. If this were true, then the central Hox genes evolved within the Bilateria, and no gene loss occurred in the Cnidaria. Unfortunately, the data at hand do not decisively distinguish among these plausible scenarios.

### Considerations regarding dataset construction

In contrast to the present study, Kamm and co-workers concluded that cnidarians diverged from bilaterians prior to the evolution of “a definitive Hox system” [49] and that cnidarians lack “true Hox genes” [50]. One basis for these conclusions was a phylogenetic analysis in which cnidarian homeodomains did not generally pair up with specific bilaterian Hox families (except for Hox1). The Kamm et al. dataset differs substantially from the current study, in that it only includes 41 Hox and ParaHox related homeodomains from Cnidaria and Bilateria [49]. Three different cnidarian species are represented: *Nematostella*, the colonial hydrozoa *Eleutheria*, and the scleractinian coral *Acropora*. However, several important gene families that appear to be close outgroups to the Hox/ParaHox clade were not represented: HlxB9, Gbx, Evx, Rough, and Mox. In addition, the authors did not include *Nematostella* homeodomain NVHD065 which, based on our own analyses, is clearly nested among the Hox and ParaHox genes of the Bilateria. Finally, the authors do not consider their data in a rooted framework.

**Figure 9:**
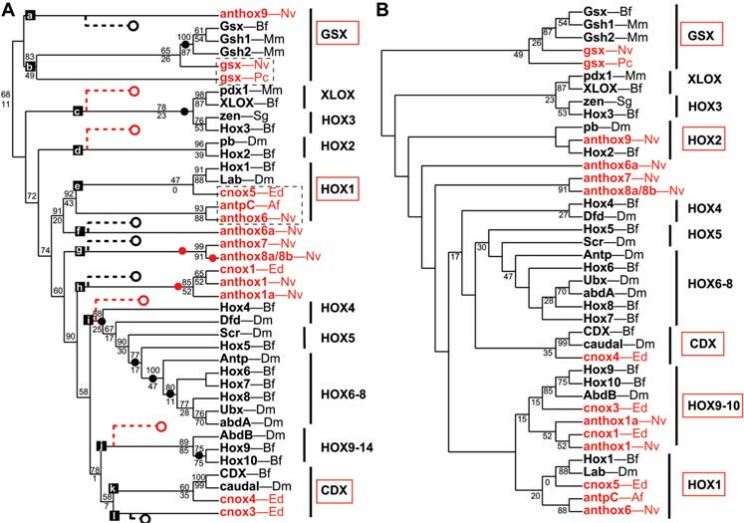
Re-analysis of the Kamm et al. maximum-likelihood phylogeny. (A) The redrawn maximum-likelihood phylogeny presented by Kamm et al. [49]. The tree is unrooted. Based on the Dayhoff-PAM1 substitution matrix [129] the tree's overall likelihood is −2288.08809. Bootstrap proportions determined in the original study are shown above the relevant nodes. Bootstrap proportions determined in the current study are shown below the relevant nodes. Inferred lineage specific gene losses are represented by open circles (black for Bilateria and red for Cnidaria). Inferred lineage specific gene duplications are represented by solid circles (black for Bilateria and red for Cnidaria). Sequences inferred to have been present in the common ancestor are indicated by lettered squares. The names of gene families that contain cnidarian representatives are enclosed by red lines. (B) A tree we identified using global instead of local rearrangements with the Kamm et al. data and the Dayhoff substitution matrix. The tree's overall likelihood is −2280.76406. Bootstrap proportions determined in the present study are shown below the relevant nodes. The dataset used in this analysis is available as Figure S13.

To determine whether differences in the composition of the two datasets might account for differences in our conclusions, we re-analyzed the Kamm et al. dataset. We used the same evolutionary model (Dayhoff) and the same phylogenetic method (maximum-likelihood) employed in the original study, but we performed a more thorough search. Where the original study performed local rearrangements on a single starting tree, we performed global rearrangements on ten randomly generated starting trees (see Methods for details). In contrast to the published study, our re-analysis groups specific cnidarian homeodomains with the Hox1, Hox2, and Hox9+ families (Figure 9). Eight out of ten trees that were identified in our analysis exhibited a higher likelihood than the single tree presented by Kamm and co-workers (Figure 1 in [49]). All eight of these trees grouped one or more cnidarian genes with Hox9–10, and three trees, including the tree with the highest likelihood (Figure 9B), grouped one or more cnidarian genes with Hox2. This result suggests that the Hox9+ family and possibly the Hox2 family were represented in the cnidarian-bilaterian ancestor.

Ironically, even if the phylogeny presented by Kamm et al. [49] accurately reflects the evolutionary relationships among cnidarian and bilaterian homeodomains, it would not support the authors' contention that the Hox system originated after the cnidarian-bilaterian split. The tree actually implies that a much more extensive Hox superfamily was present in the cnidarian-bilaterian ancestor than is currently found in either extant Cnidaria or Bilateria (Figure 9a). If the tree is rooted at Gsx (the most logical place to root their tree based on a recently published analysis of the homeodomain superclass [52]), the topology implies that the twelve distinct Hox-ParaHox genes were present in the CBA (Gsx, Hox1, Hox2, Xlox/Hox3, Hox4–8, Hox9+, and Cdx), as well as several hypothetical homeodomain lineages defined by cnidarian representatives (anthox1, anthox6a, anthox7/8a/8b, anthox9, and cnox-3). Clearly, this particular phylogeny cannot be used to argue that the Hox “system” is unique to bilaterians.

### Evolution of the Hox and ParaHox clusters

Originally it was thought that the Hox cluster was a result of a series of ancient tandem duplications [80]–[84]. In 1998, Brooke et al. [76] put forth the theory that a four-gene ProtoHox cluster (consisting of an anterior, group 3, central and posterior ProtoHox gene) duplicated to form the Hox and ParaHox sister clusters [41]. Recently several authors have proposed theories in which a two-gene ProtoHox cluster duplicated and the resulting clusters later independently underwent tandem gene duplications to form the extant Hox and ParaHox clusters [41], [51].

**Figure 10:**
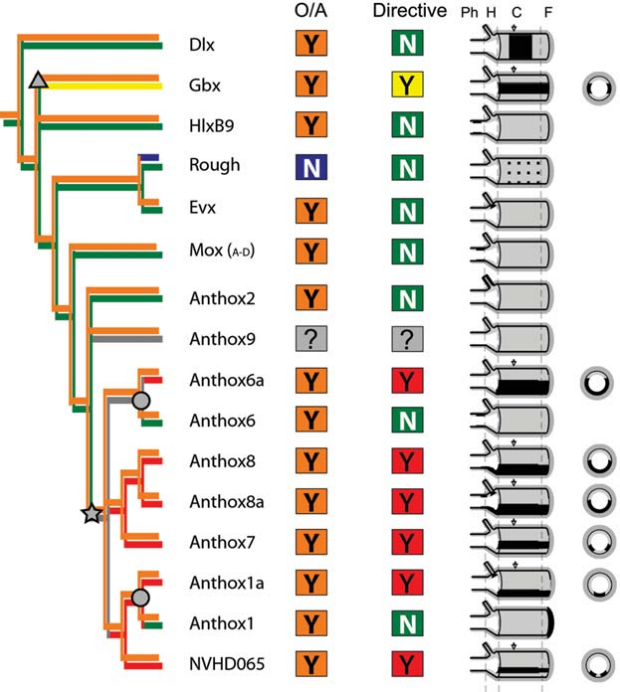
Phylogenetic mapping of Hox expression. The neighbor-joining and Bayesian phylogenies (Figure 2 and S1) were pared to remove all bilaterian sequences. The strict consensus topology shown here depicts the relative relationships among *Nematostella* sequences. Each of the *Nematostella* Hox-related sequences is coded according to whether its expression is restricted along the primary (O/A) body axis or the secondary (directive) body axis (Y = yes; N = no). A yellow Y in the directive column signifies that the expression is bilateral (both sides of the directive axis), and a red Y indicates that the expression is unilateral. The character state found in the terminal taxon is indicated in the colored boxes. The internal nodes are shaded to indicate the character states found in hypothetical ancestors. For each gene, the spatial expression is depicted on a diagram of the juvenile polyp. In the case of Dlx, anthox6a and anthox1, the expression pattern that is depicted actually occurs earlier, in the larval stage, but it is represented on a diagram of the polyp to facilitate spatial comparisons with the other genes. The polyp is drawn in lateral view with the overlying ectoderm (dark gray) partially peeled away to reveal the underlying endoderm of the body column (light gray) and the lumen of the pharynx (white). The pharynx is drawn as though everted. Only one representative tentacle is shown. The mesoglea, a largely acellular layer of connective tissue that separates the endoderm from the ectoderm, is depicted as a thin black line. Gene expression is depicted as black shading in the endoderm or ectoderm. The major regions along the primary body axis are demarcated with dotted lines: Ph = pharynx; H = head; C = column; F = foot. Cross-sectional views through the body column (at the arrowheads) are shown for Gbx, anthox7, anthox8a, anthox8b, anthox6a, anthox1a, and NVHD065.

The seven *Nematostella* Hox genes appear to trace their origins to three genes in the cnidarian-bilaterian ancestor: two anterior Hox genes and one posterior Hox gene (Figure 10). In support of the original Hox-ParaHox hypothesis [76], the neighbor-joining and Bayesian trees group the group3 Hox and ParaHox genes (Hox3, XLOX), and the neighbor-joining tree groups the posterior Hox and ParaHox genes (Hox9+, Cdx). However, none of our analyses groups Hox1, Hox2, and Gsx into a single clade as required by the original Hox-ParaHox hypothesis [76], and the mean statistical support for this relationship is 0.00 (see methods for 8 evolutionary scenarios that were tested). Furthermore, posterior Hox genes and Cdx do not form a monophyletic group in our maximum-likelihood or Bayesian analyses, and the mean statistical support for a sister-group relationship between Xlox and Hox3 is not overwhelming (0.41).

Our analysis, unlike the original Brooke et al. paper [76] and subsequent Hox/ParaHox cluster analyses [41], benefits from being able to have a full genome from which to determine reliable outgroups to root our phylogeny. The fact that Gsx forms an independent lineage in all of our analyses (Figure 2, S1, and S2), poses a major stumbling block for any theory that considers the Hox and ParaHox sister clusters.

**Figure 11:**
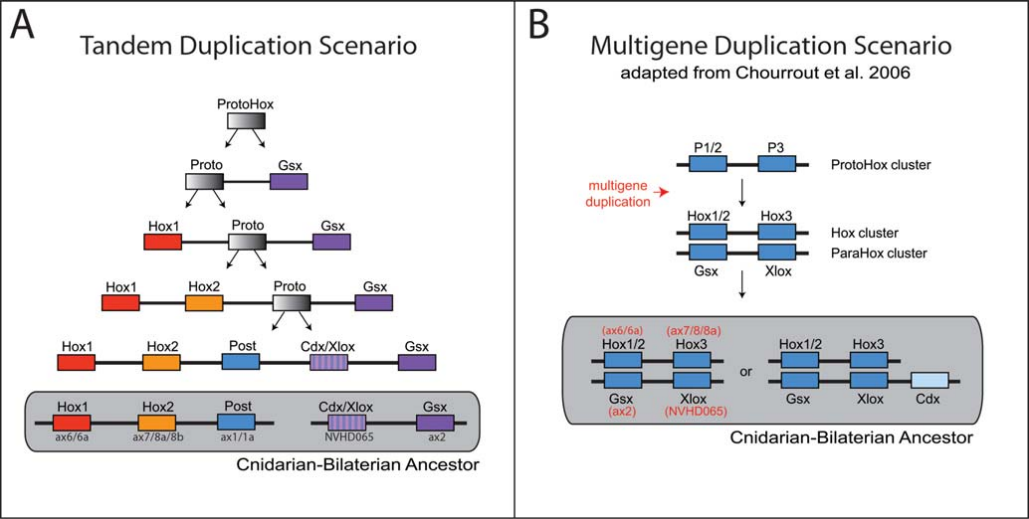
Origins of the Hox and ParaHox clusters. (A) The tandem duplication model of Hox and ParaHox clusters. Our evidence conflicts with the current theory that a multigene duplication event formed the Hox and ParaHox cluster. Under this alternative model, each Hox and ParaHox gene was formed through a series of tandem duplications either in the 3′ or 5′ direction and a translocation event separated the two ParaHox genes from the Hox cluster. Hox3 and Central Hox genes are not included in this model because it is not clear whether they were present in the cnidarian-bilaterian ancestor and subsequently lost in the Cnidaria or alternatively, were derived in the Bilateria from subsequent tandem duplication events of another Hox/ParaHox gene (for example anthox7/8a/8b or anthox1/1a). The general scenario presented here is robust enough to easily accommodate either event. (B) In this scenario adapted from Chourrout et al. 2006 Figure 3, a two-gene ProtoHox cluster is duplicated to form two sister clusters, the Hox and ParaHox clusters [51]. Chourrout et al. found that NVHD065 (Xlox/Cdx in their analysis) showed conflicting affinities for Xlox and Cdx; similarly our neighbor-joining and Bayesian trees had NVHD065 grouping with Cdx while the maximum-likelihood tree had NVHD065 grouping with Xlox. Unlike our study, Chourrout et al. considered anthox1 and anthox1a (HoxE and HoxF in their analysis) to be non-Hox/ParaHox lineages, hence their omission from their model [51].

Several lines of evidence in our data suggest a novel hypothesis—that this two-gene ParaHox cluster may have been formed as a result of a tandem duplication rather than a cluster duplication. First and foremost, Gsx consistently emerges as an independent lineage, which is inconsistent with a cluster-duplication scenario, and characteristic of a lineage that was formed by a tandem duplication event. Also, a Hox3 gene has never been recovered from a cnidarian despite numerous PCR surveys (for example [66], [67], [71]), EST studies (for example [85], [86] ) and two full genome scans (*Hydra* [51] and *Nematostella* [49]–[52]). The moderate affinity of Hox3 for Xlox in our analyses may be due to convergence or it may represent a bilaterian-specific duplication. Finally, any evidence uniting Cdx and posterior Hox genes or Hox3 and Xlox could simply have been a result of those genes being the last in a series of tandem duplications. It is not difficult to imagine a scenario in which a series of tandem duplication events, occurring at both the 5′ and 3′ ends of a primordial ProtoHox cluster, could have led to the ParaHox genes Gsx and NVHD065 being clustered adjacent to one another at one end of the cluster and, subsequently, detached via a translocation event (Figure 11I). If this scenario is correct, the ParaHox genes would represent detached Hox genes rather than a sister cluster.

### Do cnidarians employ a Hox code?

To say that cnidarians utilize a Hox code, as this concept is generally understood, they must possess multiple Hox genes, and these genes must specify distinct regions along a body axis. Most authors refer specifically to the primary body axis when discussing the Hox code, but other conserved Hox axes have been described, for example in the vertebrate urogenital system, digestive system, and paired appendages [87]–[89]. Evidence suggests that the ancestor to bilaterians had a seven gene Hox cluster and at least a three-gene ParaHox cluster [41]. However, as is evident in several disparately related species, the existence of a Hox code does not require the existence of intact Hox clusters or Hox colinearity. Despite claims to the contrary [49], [50], our findings suggest that *Nematostella* has at least three “true” Hox genes and two “true” ParaHox genes, and their Hox genes are expressed in distinct regions along the primary body axis. In addition, there is clear evidence for both a Hox cluster and ParaHox cluster in *Nematostella* [51].

### Nematostella Hox Linkage and the “Hox-code”

Recently, Kamm and co-workers [49] concluded that, “with the exception of independently duplicated genes, the cnidarian [Hox-related] genes are unlinked.” Unfortunately, their study relied on contigs generated by searching the NCBI trace archive with MegaBlast rather than a full genome assembly, and this conclusion proved to be incorrect [49]. With the benefit of additional data from two publicly available genome assemblies and gene mapping experiments, we can clearly recognize linked clusters of Hox and Hox-related genes that were not detected by Kamm et al. [49]. Our linkage findings corroborate those of Chourrout and co-workers [51] who used another genome sub-assembly method, described only by name (Marche à Droite (Màd)), to produce their own set of *Nematostella* contigs.

### Nematostella Hox gene expression and the “Hox-code”

The seven putative Hox genes of *Nematostella* are expressed in three principal domains along the primary body axis and, collectively, these account for practically the entire axis: the pharynx, the body column, and the aboral extremity. Anthox6 is expressed in an endodermal ring lining of the pharynx at the oral end of the body, adjacent to *Nv-otx* expression [90]. Anthox1a, anthox7, anthox8a, and anthox8b are expressed widely throughout the endoderm of the body column, from the tentacle zone to the foot region. Anthox1 is expressed in a discrete spot of ectoderm at the aboral extremity.

We hypothesize that multiple Hox genes are involved in patterning the primary body axis of *Nematostella*. This conclusion is clearly supported by the expression data, though functional studies will be necessary to prove that Hox genes are *required* for axial patterning in *Nematostella*. If Hox genes are involved in patterning the primary body axis of *Nematostella*, this suggests that a simple “Hox code” existed prior to the divergence of Cnidaria and Bilateria. To presume that distinct Hox expression domains have independently evolved to differentiate axial regions along the primary axis in both the Cnidaria and the Bilateria is clearly less parsimonious. Importantly, we should not expect a cnidarian “Hox code” to be as elaborate as that seen in the model bilaterians because the Cnidaria have not achieved the same degree of axial complexity as fruit flies or vertebrates. Nor should we expect a precise correspondence between the Hox expression territories of Cnidaria and Bilateria, as independent evolution within the long diverged cnidarian and bilaterian lineages will tend to obscure evidence of homologous pattering mechanisms. Furthermore, to say that a Hox code existed prior to the cnidarian-bilaterian split is not to say that the Hox code was the original metazoan axial patterning system. Other possible axial patterning systems, such as the Wnt genes [91], may have been in place prior to the Hox code. Studies on additional basal metazoan taxa may shed light on the origin of animal body axes.

Kamm and coworkers [49] have argued against the existence of a cnidarian Hox code because the larval expression of *Nematostella* anthox6 differs from its ortholog in the colonial hydrozoan Eleutheria [49]. However, the Anthozoa and Hydrozoa are separated by more than 500 million years of evolutionary time [92], and the compound life history of the Hydrozoa, which features two major adult body plans (polyp and medusa), is believed to be a derived feature within the Cnidaria [93]. Furthermore, the polyp of medusozoan cnidarians (including Hydrozoa), are believed to be secondarily reduced [93]. It would not be surprising if the origin of a novel adult body plan, accompanied by the simplification of the ancestral adult body plan, would be associated with changes in the spatial deployment of Hox genes. The origin of novel body plans has been accompanied by radical alterations of Hox expression in both echinoderms [94] and cephalopods [95]. In addition, Hox gene expression patterns are known to change radically even in the absence of major alterations to animal body plans; for example, the Hox3 homologs of *Drosophila* have abandoned their ancestral role in the Hox code [96]–[100].

Kamm and coworkers [49] argue that the expression of *Nematostella*'s Hox genes in different germ layers is additional evidence that the “Hox system” originated after the cnidarian-bilaterian split. It is quite common for Hox genes to be expressed in multiple germ layers in the Bilateria. For example, while Hox genes are most often thought of as being expressed in the central nervous system [1], [7], [8], [101], [102], Hox genes are also coordinately expressed in axial [3], [103] and limb bud mesoderm [104], as well as in endodermal derivatives [9], [10], [105], [106]. Interestingly, Hox gene expression in mesoderm has been shown to be important for endodermal patterning [107] and endodermal expression of Hox genes in anthozoans may be related to the bifunctional mesendodermal status of anthozoan gastrodermis [108].

Ironically, despite Kamm and coworkers [49] using the expression patterns of “Hox-like” genes in the hydrozoan *Eleutheria* as counter evidence for the existence a “Hox system”, the genes show clear signs of axial expression [49]. The “posterior Hox/Cdx-like” gene Cnox-4ed is expressed at the aboral pole (Figure 3H in [49]) and the “anterior Hox-like” gene Cnox-5ed is expressed at the opposite oral pole of primary polyps (Figure 3E and 3M in [49]). This evidence suggests that at least some components of the “Hox system” are evident in this highly derived hydrozoan.

In contrast to Kamm et al., we have the advantage of surveying the expression of the entire compliment of *Nematostella* Hox genes. One interesting feature gleaned from this survey is the potential role of *Nematostella* Hox genes in patterning the secondary (directive) body axis. Anthox1a shows a highly restricted pattern along the ventral midline of the endodermal body wall (Figure 5J) and anthox7 shows a complimentary pattern on its dorsal boundary (Figure 5N), while anthox8a and anthox8b encompasses the entire anthox1a and anthox7 expression domain (Figure 5J–5U). Anthox6a shows the broadest domain of expression, extending across approximately 2/3 of the dorso-ventral axis (Figure 5E). Only the midline of the dorsal body wall fails to express Hox genes, although the dorsal pharynx expresses anthox8a (Figure 5P and 5R).

### Evolution of axial patterning

Considering the evidence presented here, it appears possible that both the Cnidaria and the Bilateria utilize Hox genes to pattern both the primary and secondary body axes. It is therefore interesting to speculate which role evolved first. Mapping the expression patterns of the Hox-related genes of *Nematostella* onto the neighbor-joining phylogeny suggests that restricted expression along the primary body axis is a primitive trait of the larger Hox-related clade (Figure 10). Of the basal Hox-related lineages (Gbx, HlxB, Rough, Eve, Mox) all exhibit restricted expression along the primary body axis except for Rough. None exhibit restricted expression along the secondary axis, except for Gbx. In contrast, the majority of the genes that make up the Hox and ParaHox clade exhibit restricted expression along the secondary body axis. Additionally, the restricted expression of Gbx along the secondary axis differs from the restricted expression of Hox/ParaHox genes along the same axis in that Gbx expression is not offset to the ventral side. Gbx is expressed in a broad swath in the middle of the directive axis.

The most parsimonious explanation for these data is that restricted expression along the directive axis evolved twice in *Nematostella* Hox genes, once in the Gbx lineage (see triangular node on Figure 10), and once near the base of the Hox-ParaHox radiation (see star-shaped node on Figure 10). A less parsimonious competing hypothesis would be that directive patterning was lost in three lineages (Rough/Eve, Mox, and anthox2) and then became restricted to a single side of the embryo in the Hox/ParaHox lineage.

Regardless of which of these hypotheses is favored, the analysis suggests that the ancestral gene that gave rise to all Hox and ParaHox genes (besides Gsx) might have been differentially expressed along both the primary and secondary body axis. Later radiations of this ancestral gene led to (at least) four main lineages: anthox6/6a, anthox7/8a/8b, anthox1/1a, and NVHD065. At least three of these ancestral genes (*i.e.*, anthox6/6a, anthox7/8a/8b, and anthox1/1a) subsequently radiated independently in the lineage leading to *Nematostella*. In the anthox6/6a and anthox1/1a lineages (see circular nodes on Figure 10), the directive-patterning component would appear to have been lost in one of the resulting paralogs (i.e. anthox6 and anthox1). While this analysis is sensitive to tree topology, it offers novel predictions about the evolution of Hox-gene expression. A thorough test of these predictions will require expression data for the full complement of Hox-related genes from other basal animal models, including other Anthozoa, medusozoan cnidarians, ctenophores, and sponges.

### Conclusions

Cnidarians (in particular, anthozoan cnidarians) have the potential to provide important insight into the evolution of metazoan body plans. Although not morphologically complex, these animals have a surprisingly sophisticated genomic repertoire that might link the axial organization of anthozoans with bilaterians. The current multi-dimensional study takes an in-depth look at the phylogenetic relationships, patterns of gene expression, and genomic arrangements of Hox genes and ParaHox genes of the starlet sea anemone, *Nematostella vectensis*. Our results demonstrate the following. (1) *Nematostella* has a seven-gene cluster comprising three *bona fide* Hox genes and four Hox-related genes and a separate two-gene ParaHox cluster (previously reported in [51]). (2) There appear to be seven *bona fide* Hox genes in *Nematostella* belonging to the Hox1, Hox2, and Hox9–14 subfamilies, and they are expressed in three distinct domains that collectively span almost the entire primary body axis. The expression of multiple Hox genes in distinct domains along the primary body axis suggests the existence of a rudimentary “Hox code.” (3) Hox genes are also expressed in nested spatial domains along the secondary (directive) axis, which suggests that they could be playing a role in patterning this body axis. (4) Phylogenetic evidence suggests that the Hox and ParaHox clusters may have formed through a series of independent tandem gene duplications and not necessarily via a cluster duplication. Additional data from other basal metazoan groups will help to elucidate which of *Nematostella's* traits are ancestral and which might be derived. The additional taxa that should prove especially useful would be (1) another anthozoan cnidarian such as a scleractinian coral, a sea pen, or a ceriathian tube anemone: (2) one or more medusozan cnidarians such as *Hydra*, *Hydractinia*, *Eleutheria*, or *Aurelia*; (3) a ctenophore, such as *Mnemiopsis*; (4) the placozoan *Trichoplax*; (5) a sponge, such as *Amphimedon* (formerly *Reniera*); (6) and an acoel flatworm.

## Materials and Methods

### Phylogenetic Analysis

The evolutionary relationships among 61 Hox-related genes were estimated by performing three separate phylogenetic analyses (neighbor-joining [77], Bayesian inference [109], [110], and maximum-likelihood [111] on the 60 amino acids of the homeodomain (Figure 1). The dataset utilized in this study is as compact as possible to minimize computer run times while still representing all of the known Hox-related gene families of Cnidaria, Deuterostomia, and Protostomia. To represent cnidarians, eighteen Hox-related genes were chosen from the sea anemone *Nematostella vectensis*. These Hox-related genes were identified in a previous study that compared complete or near-complete Hox complements from the sequenced genomes of human, fruit fly, *Nematostella* [52]. Unlike this previous study, we selected 20 homeodomains from the lancelet *Branchiostoma floridae* and only one homeodomain from *Homo sapiens* to represent the deuterostomes. In general, the lancelet was preferred over the human because its lineage has not experienced the Hox cluster duplications that have occurred during the ancestry of vertebrates [112]. However, a human sequence was chosen to represent the HlxB9 gene family because HlxB9 has not yet been recovered in *Branchiostoma*. To represent the protostomes, we selected 16 homeodomains from *Drosophila melanogaster,* one from the spider *Cupiennius salei* [*113*], and one from the polychaete worm *Capitella* [106]. The Hox3 gene of *Cupiennius* was selected because the three Hox3-related genes of Drosophila (*zen1*, *zen2*, and *bicoid*) are known to be highly derived relative to the ancestral Hox3 sequence [96]–[100]. The XLOX homeodomain of *Capitella* was selected because XLOX has not been identified in the fruitfly. Distalless (dll) sequences from *Nematostella*, *Branchiostoma*, and *Drosophila* were used as an outgroup—this outgroup designation is based on the results of a much more extensive previous analysis [52]. The phylogenetic dataset is available as a supplemental file (Figure S12).

Neighbor-joining [77] analysis was performed using the computer package Phylip (version 3.6.1; [114]). Distances among homeodomains were calculated using the ProtDist program of Phylip and the James-Taylor-Thorton (JTT) distance matrix [115]. The JTT matrix was determined to be superior to the other available distance matrices within ProtDist using ProtTest 1.3 [116]. Support for clades on the neighbor-joining tree was assessed by 1000-replicates of bootstrap [117]. The neighbor-joining tree, like the other two trees, was re-drawn and re-rooted using the Dll sequences as an outgroup with the computer program MacClade, version 4.03 [118].

Maximum-likelihood [111] analysis was performed using the Proml program of the computer package Phylip, version 3.6.1 [114]. The best model of protein evolution available within Proml was determined using ProtTest 1.3 [116]: JTT plus Gamma distributed rates plus invariant residues. Five rate categories were specified, including one category to accommodate invariant residues. The proportion of invariant residues was determined empirically (0.119). The coefficient of variation of substitution rates among sites was set to 0.893. Global rearrangements were performed on 1000 starting trees produced by random addition of taxa. Support for particular clades was assessed by 100-replicates of the bootstrap [117].

Bayesian analysis was performed using MrBayes version 3.1.2-MPI under a mixed rate model (aamodelpr = mix) [119]. Two simultaneous, completely independent Markov chain Monte Carlo searches were run, starting from different random trees (Nruns = 2). The search was conducted for 20,000,000 generations with trees being sampled every 100 generations and printed every 1000. Each Markov chain generated 200,000 trees. The two resulting treefiles were meshed and the first 80,000 trees were discarded as “burnin.” The Consense program of Phylip [114] was used to build a “Majority rule (extended)” tree from the remaining 320,000 trees.

To facilitate direct comparisons among studies, we also re-analyzed the homeodomain dataset of Kamm et al. [49]. In our re-analysis of the Kamm et al. dataset, we used the same basic phylogenetic methodology that generated Figure 1 of the original study: we performed a maximum-likelihood analysis using the Dayhoff amino acid substitution matrix. However, where the original study utilized local rearrangements on a single starting tree, we conducted a more thorough search using global rearrangements on ten randomly generated starting trees. The phylogenetic dataset is available as a supplemental file (Figure S13).

### Statistical support for alternative hypothesis

Statistical support for alternative hypotheses was determined by constructing a constraint trees for each alternate hypothesis and using the constraints to filter trees from each analysis (100 bootstrap trees from maximum-likelihood, 1000 trees from neighbor-joining analysis, and 320,000 trees from Bayes) in Paup* [120]. The mean statistical support is the average of the neighbor-joining bootstrap proportion, the Bayesian posterior probability, and the maximum-likelihood bootstrap proportion. It is expressed as a percent of trees in which the given grouping was recovered. Because of rounding, 0.00 does not mean absolutely no support, rather it means less than 0.05.

For the relationship of Gsx genes with the Hox1 and Hox2 lineages, 8 constraints with various *Nematostella* members were constructed. The bilaterian Hox1, Hox2, and Gsx genes and the *Nematostella* gene anthox2 were the core members of each constraint. One set of analysis included anthox6 and anthox6a in addition to the core members. Another set included anthox7, anthox8a, and anthox8b along with anthox6 and anthox6a. In addition, anthox2a was added to each of the previous constraints.

### Determination of Gene Linkage and Gene Structure

Clusters of Hox-related homeobox genes were identified by screening a draft assembly of the *Nematostella* genome with a dataset of 123 *Nematostella* homeobox sequences [52]. The assembly was produced using the Phusion program [121] and is available at StellaBase [53], [54] Clusters were verified and extended by comparison with the genome assembly produced by the Joint Genome Institute [55]. Additional experimental confirmation was provided by the following: (1) isolation and sequencing of clones from a genomic library prepared in the Lambda FixII vector (Stratagene), (2) genomic walking using the techniques of ligation-mediated PCR and long PCR, and (3) isolation and sequencing of transcripts obtained by screening a cDNA library or by RACE. Comparisons between the transcripts and the genomic sequence were used to determine gene structure.

### Isolation of transcripts

Gene specific primers were designed to amplify partial transcripts using 5′ and 3′ RACE. Where possible, full-length transcripts were assembled by conceptually splicing overlapping 5′ and 3′ RACE products. Primer design was based on smaller gene fragments originally obtained by degenerate PCR in the case of anthox1, anthox1a, anthox7, anthox8, and evx, [44], [45]. Full length transcripts were isolated from a cDNA library for anthox2 and anthox6 [46]. Primer design was based on consulting genomic trace sequences for Dll, HlxB9, Gbx, Mox, Rough, anthox2a, anthox9, and NvHD065. All sequences have been deposited in the GenBank database (Accession numbers are provided in Figure 1.). Primer sequences are available upon request from M. Q. Martindale (mqmartin@hawaii.edu)

### In Situ Hybridization[108]

For each gene under study, digoxygenin-labeled sense and anti-sense probes were produced using the 3′ RACE products as templates according to published protocols [57], [62], [108], [122]. All probes spanned a small region of the homeobox, the coding sequence downstream of the homeobox and the 3′ untranslated region. Probe length ranged from 323–1200 nucleotides. Hybridizations were performed as previously described at 65°C for 20–44 hours at probe concentrations of 1.0 ng/ml.

### Phylogenetic analysis of gene losses, gene gains, and gene expression changes

Gene expression patterns for 14 *Nematostella* genes were scored with respect to whether their expression is axially restricted along the primary axis, the oral-aboral axis, and the secondary axis, the directive axis [123]. These character states were then mapped onto neighbor-joining, Bayesian, and maximum-likelihood phylogenies of the 14 *Nematostella* genes. The phylogenies were obtained by removing non-*Nematostella* taxa from the neighbor-joining analysis in figure 2, the Bayesian phylogeny in supplemental figure 1, and the maximum-likelihood phylogeny in supplemental figure 2. Character mapping was performed using the ACCTRAN character state optimization method implemented in the computer program MacClade, version 4.03; [124].

## Supporting Information

Figure S1 Homeodomain phylogeny based on Bayesian inference. The tree is rooted and labeled in the same manner as Figure 2. Posterior probabilities are shown at each node. The dataset used in this analysis is available as Figure S12.
(1.27 MB EPS)

Figure S2 Homeodomain phylogeny based on maximum-likelihood. The tree is rooted and labeled in the same manner as Figure 2. Bootstrap proportions are shown at each node. The overall likelihood of the tree is −3103.08424. The dataset used in this analysis is available as Figure S12.
(1.27 MB EPS)

Figure S3 Annotated anterior-Hox cluster of *Nematostella*. Two overlapping genomic scaffolds (C408300840.Contig2 and C402800703.Conting1) that contain seven ANTP class genes (anthox6, anthox7, anthox8a, anthox8b, Evx, Rough, and HlxB9) were identified in an assembly of the *Nematostella* genome produced using the computer program Phusion [121]. A PRD class homeobox belonging to the Dmbx family is also linked to this cluster. These eight homeobox genes are clustered within a 329,244-nucleotide span. A 4,930-nucleotide region containing part of the second exon of anthox8b is common to both genomic scaffolds (double dotted line). The cluster was confirmed computationally by comparing it to the independently generated *Nematostella* genome assembly produced by the Joint Genome Institute (U. S. Department of Energy) [55]. The two overlapping genomic scaffolds in the Phusion assembly align to a single scaffold in the JGI assembly spanning 1073713 nucleotides (scaffold-61). The larger JGI scaffold also encompasses an additional ANTP class homeodomain belonging to the HLX family (HLXd; [52]). Portions of the cluster were corroborated experimentally through the independent sequencing of RACE products, cDNA clones, genomic clones, and PCR fragments. The locations of these corroborating segments are indicated below the cluster. The sequence of the anthox7, anthox8a, anthox8b, Evx, Rough, and HlxB9 transcripts were determined by conceptually splicing overlapping 5′ and 3′ RACE products. The sequence of the anthox6 transcript was previously determined by isolating and sequencing a full-length clone from a cDNA library [46]. Transcriptional orientation is indicated by an arrow above each locus. Coding regions of homeobox genes are indicated by boxes: red = homeobox sequences; gray = other protein coding regions; black = untranslated regions.; Gray spheres indicate approximate location of non homeodomain genes predicted from BLASTx search of the RefSeq database (NCBI: latest release_07.17.06). Lengths of segments flanking homeodomain genes (in nucleotides) are given.
(1.09 MB EPS)

Figure S4 Annotated Mox cluster of *Nematostella*. Four Mox loci exhibiting the same transcriptional orientation (5′ to 3′ from left to right), are linked within a span of 23,212 nucleotides on a single genomic scaffold in the Phusion assembly 112,563 nucleotides long. A 345,744 nucleotide scaffold encompassing this cluster is contained in the JGI assembly of the *Nematostella* genome (scaffold-248) [55]. A TBLASTN analysis of the scaffold against the homeodomain dataset did not identify any additonal homeodomains. The sequence of moxD and moxC transcripts were determined by conceptually splicing overlapping 5′ and 3′ RACE products. 3′ RACE products were used to annotate the homeodomain containing exon and the 3′ UTR of moxB and moxA. The figure is labeled as in Figure S3.
(0.63 MB EPS)

Figure S5 Annotated ParaHox cluster of *Nematostella*. The Gsx ortholog, anthox2 is linked to the possible Cdx/Xlox homolog, NVHD065. A single genomic scaffold (402901197.Contig1) containing the Gsx ortholog and NVHD065 was identified in an assembly of the *Nematostella genome* produced using Phusion [121]. The structures of Gsx and NVHD065 were coroborated experimentally through independent sequencing of RACE products and cDNA clones. The sequence of the Gsx transcript was previously determined by isolating and sequencing a full-length clone from a cDNA library[46], while the NVHD065 transcript was determined by conceptually splicing overlapping 5′ and 3′ RACE products. A 1,580,679 nucleotide scaffold encompassing this cluster is contained in the JGI assembly of the *Nematostella* genome (scaffold-27) [55]. A TBLASTN analysis of the scaffold against the homeodomain dataset did not identify any additional homeodomains. The figure is labeled as Figure S3.
(0.59 MB EPS)

Figure S6 Annotated anthox1a cluster of *Nematostella*. Two Hox family genes were mapped to a single 98,121 nucleotide genomic scaffold in the Phusion assembly (c401800829.Contig2). The sequence of the anthox 9 transcript was determined by conceptually splicing overlapping 5′ and 3′ RACE fragments while the sequence of anthox1a was previously determined by isolating and sequencing a full-length clone from a cDNA library which was further extended using ligation mediated PCR. A 2,832,129 nucleotide scaffold (scaffold-03) was recovered from the JGI assembly that encompassed the Phusion scaffold [55]. The anthox9 - anthox1a locus begins approximately 58,8435 nucleotides from the start of the scaffold. A subsequent TBLASTN analysis of the JGI_03 scaffold against the homeodomain database recovered three additional ANTP class genes. MsxlxA and MsxlxB were mapped approximately 343,185 bases downstream of anthox1a and are separated from each other by approximately 8945 nucleotides. An EST (DV089324) was recovered from the NCBI database that mapped to the MsxlxA homeodomain. An HLXc-like homeodomain was identifed approximately 1,809,900 nucleotides away from MsxlxB. The figure is labeled as Figure S3.
(0.63 MB EPS)

Figure S7 Annotated anthox1-containing genomic scaffold of *Nematostella*. Anthox1 was identified on a single 359,083 nucleotide scaffold in the Phusion assembly (c407500859.Contig1). A previously recovered 5′ RACE product that was further extended using ligation-mediated PCR was used to determine the sequence of anthox1. This RACE product was used to probe the *Nematostella* genomic library and a single genomic clone was identified and sequenced (AA8a). A 2,783,717 nucleotide scaffold (scaffold-04) was recovered from the JGI assembly that encompassed the Phusion scaffold [55]. The anthox1 locus begins approximately 1,711,804 nucleotides from the start of the scaffold. A subsequent TBLASTN analysis of the JGI_04 scaffold against the homeodomain database recovered three additional ANTP class genes. Emxlx is located approximately 549,540 nucleotides, and HLXa approximately 306,200 nucleotides upstream of anthox1, while an ANTP class ambiguous family homeodomain is approximately 434,972 nucleotides downstream of anthox1. The figure is labeled as Figure S3.
(0.61 MB EPS)

Figure S8 Annotated anthox6a cluster of *Nematostella*. Anthox6a and HLXb were identified from a single genomic scaffold (c403601169.Contig1) from the Phusion assembly of the *Nematostella* genome. The sequence of anthox6a and HLXb was determined by conceptually splicing overlapping 5′ and 3′ RACE products. A 1,571,464 nucleotide scaffold encompassing this cluster is contained in the JGI assembly of the *Nematostella* genome (scaffold-26) [55]. A TBLASTN analysis of the scaffold against the homeodomain dataset did not identify any additional homeodomains. The figure is labeled as Figure S3.
(0.59 MB EPS)

Figure S9 Annotated Gbx cluster of *Nematostella*. Gbx was localized to a single 117,312 nucleotide genomic scaffold (c402901116.Contig1) from the Phusion assembly of the *Nematostella* genome. The sequence of Gbx was determined by conceptually splicing overlapping 5′ and 3′ RACE products. A 1,068,422 nucleotide scaffold encompassing this cluster is contained in the JGI assembly of the *Nematostella* genome (scaffold-58) [55]. A TBLASTN analysis of the scaffold against the homeodomain dataset identified an additional homeodomain, HLXc located approximately 601,580 nucleotides upstream of Gbx. The figure is labeled as Figure S3.
(0.59 MB EPS)

Figure S10 Annotated Dlx-NVHD021 cluster of *Nematostella*. Dlx and NVHD021 are located 6988 nucleotides apart on a single genomic scaffold (c413501012.Contig1) from the Phusion assembly of the *Nematostella* genome. The sequence of Dlx was was determined by conceptually splicing overlapping 5′ and 3′ RACE products, while the longest open reading frame downstream of NVHD021 homeodomain was inferred using MacVector 7.2.3 (Accelrys Inc.). A 1,601,021 nucleotide scaffold encompassing this cluster is contained in the JGI assembly of the *Nematostella* genome (scaffold-20) [55]. A TBLASTN analysis of the scaffold against the homeodomain dataset did not identify any additional homeodomains. The figure is labeled as Figure S3.
(0.58 MB EPS)

Figure S11 Expression of MoxB, MoxC, and MoxD in *Nematostella*. *In situ* hybridization reveals the spatial expression of MoxA (A–D), MoxC (E–F), and MoxD (G–H) during larval development. Photos F and H depict optical cross-sections through the primary body axis, the oral-aboral axis, at the level of the developing pharynx. All other photos shown are optical longitudinal sections along the principal body axis. The blastopore, which will give rise to the mouth, is indicated by an asterisk. The division between ectoderm (ec) and endoderm (en) is clearly visible in all photos. At later stages (for example, panel D), body wall endoderm (enbw) can be distinguished from pharyngeal endoderm (enph). Tentacles (tn) emerge around the blastopore/mouth at the late larval stage (visible in C, D, and G). Prior to settlement at approximately 5–7 days, the first two mesenteries (mes) and pharynx (pha) have developed, and the gastrovascular cavity, or coelenteron (coe), is clearly visible (D).
(4.63 MB EPS)

Figure S12 Alignment of homeodomains used in our phylogenetic analysis in phylip format.
(0.73 MB EPS)

Figure S13 Alignment of homeodomains used in the re-evaluation of Kamm et al. dataset in phylip format [49].
(0.45 MB EPS)
